# Trends in Effectiveness of Organizational eHealth Interventions in Addressing Employee Mental Health: Systematic Review and Meta-analysis

**DOI:** 10.2196/37776

**Published:** 2022-09-27

**Authors:** Elizabeth Stratton, Amit Lampit, Isabella Choi, Hanna Malmberg Gavelin, Melissa Aji, Jennifer Taylor, Rafael A Calvo, Samuel B Harvey, Nick Glozier

**Affiliations:** 1 Central Clinical School Faculty of Health and Medicine University of Sydney Camperdown Australia; 2 ARC Centre of Excellence for Children and Families over the Life Course Sydney Australia; 3 Academic Unit for Psychiatry of Old Age, University of Melbourne Parkville Australia; 4 Department of Psychology Umeå University Umeå Sweden; 5 Dyson School of Design Engineering Imperial College London London United Kingdom; 6 School of Psychiatry, University of New South Wales Sydney Australia; 7 Black Dog Institute Sydney Australia; 8 St George Hospital Sydney Australia

**Keywords:** eHealth, mental health, employee, systematic review, mobile phone

## Abstract

**Background:**

Mental health conditions are considered the leading cause of disability, sickness absence, and long-term work incapacity. eHealth interventions provide employees with access to psychological assistance. There has been widespread implementation and provision of eHealth interventions in the workplace as an inexpensive and anonymous way of addressing common mental disorders.

**Objective:**

This updated review aimed to synthesize the literature on the efficacy of eHealth interventions for anxiety, depression, and stress outcomes in employee samples in organizational settings and evaluate whether their effectiveness has improved over time.

**Methods:**

Systematic searches of relevant articles published from 2004 to July 2020 of eHealth intervention trials (app- or web-based) focusing on the mental health of employees were conducted. The quality and bias of all studies were assessed. We extracted means and SDs from publications by comparing the differences in effect sizes (Hedge *g*) in standardized mental health outcomes. We meta-analyzed these data using a random-effects model.

**Results:**

We identified a tripling of the body of evidence, with 75 trials available for meta-analysis from a combined sample of 14,747 articles. eHealth interventions showed small positive effects for anxiety (Hedges *g*=0.26, 95% CI 0.13-0.39; *P*<.001), depression (Hedges *g*=0.26, 95% CI 0.19-0.34; *P*<.001), and stress (Hedges *g*=0.25, 95% CI 0.17-0.34; *P*<.001) in employees’ after intervention, with similar effects seen at the medium-term follow-up. However, there was evidence of no increase in the effectiveness of these interventions over the past decade.

**Conclusions:**

This review and meta-analysis confirmed that eHealth interventions have a small positive impact on reducing mental health symptoms in employees. Disappointingly, we found no evidence that, despite the advances in technology and the enormous resources in time, research, and finance devoted to this area for over a decade, better interventions are being produced. Hopefully, these small effect sizes do not represent optimum outcomes in organizational settings.

**Trial Registration:**

PROSPERO CRD42020185859; https://www.crd.york.ac.uk/prospero/display_record.php?RecordID=185859

## Introduction

Mental health conditions are to be considered the leading cause of disability, sick leave, and long-term work incapacity in most developed countries [1]. Furthermore, poor mental health has a substantial impact on employee well-being, productivity, absenteeism, compensation claims, and social welfare systems [2,3]. Evidence supports the increased demand for workplace interventions, highlighting that working conditions and the workplace environment can influence employees’ mental health and well-being [4].

The nature of mental health symptoms fluctuates on a continuum between thriving and struggling [5]. Most mental ill health that is seen in the workforce is because of common mental disorders, most notably, depression, anxiety, and stress-related conditions [1,6,7]. Employees who have a mental health condition and become too unwell to continue working rarely move straight from being *healthy* to needing sick leave [8]. There is usually a course that an employee might experience as they develop worsening symptoms [9], and different interventions may be required at different stages of this course.

International approaches suggest multilevel organizational approaches targeting (1) healthy workers via universal prevention interventions; (2) those with subclinical conditions (symptomatic or at-risk workers), such as those experiencing high stress via indicated interventions; and (3) those workers who have disclosed a mental health condition with tertiary interventions (treatments) [8]. Furthermore, the UK Thriving at Work Review into Mental Health at Work recommends the inclusion of the use of therapeutically tailored interventions based on individual-specific needs [5].

The 2 potential rate-limiting steps for organizations in implementing mental health intervention programs are the budget and logistics of delivering universal interventions to all staff at scale and the ability to target indicated and early interventions to those who are at risk or unwell before disclosure. The internet offers a unique opportunity to address these rate-limiting steps by delivering eHealth interventions with components such as cognitive behavioral therapy (CBT) and stress management to a broad audience. eHealth interventions provide employees with access to psychological help when they are not employed in a typical working environment, such as shift workers or those working from home, or when they may be sick-listed from work. As a result, eHealth interventions have been widely implemented and provided. eHealth is an emerging field in public health and business and provides health services and information delivered or enhanced through the internet and related technologies, such as smartphone apps. Recently, eHealth has been seen as a popular approach in organizations as it provides an inexpensive and anonymous way of addressing common mental disorders [10], including apps linked to wearable devices, and guided meditation programs [11].

There is a plethora of evidence for the short- and long-term benefits of eHealth-delivered CBT for treating anxiety and depressive conditions in both the general population and clinical settings [12-15]. Evidence has also emerged for the effectiveness of mindfulness-based eHealth interventions in improving symptoms for both the general population and individuals who are symptomatic [16].

However, we know that employed individuals differ systemically from both general and clinical populations used in most eHealth studies; for instance, employees have much better mental health (fewer symptoms) than general and clinical populations [17], for whom many eHealth interventions and their content have been developed. As such, there are likely to be floor effects and other efficacy modifiers. The delivery in, and by, organizations will be different from just open access to interventions in the general population. This influences uptake and engagement, which are known determinants of digital health efficacy [18].

A previous meta-analysis considering eHealth interventions from 2004 (first identified) to 2017 [19] found randomized controlled trial (RCT) evidence for only 23 eHealth interventions delivered to employees. Overall, there was a small pooled effect of reducing depression, anxiety, and stress symptoms after the intervention (Hedges *g*=0.24), which was sustained at follow-up (Hedges *g*=0.23). Similar effect sizes (ESs) were found in a contemporaneous review [20]. Only 2 years later, a meta-analysis [21] identified 50% (n=34) more studies of such interventions and suggested stronger effects on stress (Hedges *g*=0.54), insomnia (Hedges *g*=0.70), and burnout (Hedges *g*=0.51) but not symptoms of depression or anxiety.

None of the previous reviews have addressed the key issue of efficacy, which is affected by the *digital placebo effect*. Smartphone app users can experience significantly reduced mental health symptoms, even if the app does not contain any direct therapeutic intervention, similar to the placebo effect seen in pharmaceutical trials when using “active” or “attention” controls [22], or may reflect regression to the mean of fluctuating symptoms. Even simply tracking the symptoms of depression in an app can lead to significant reductions in depression [23]. Both processes result in an apparent “intra-group” effect without any therapeutic intervention. In a trial, the type of control used is a primary determinant of the “between-group” ES. In a recent systematic review of smartphone apps for anxiety in clinical samples, the between-group ESs were lower in trials that used active controls than in those that used passive controls [24]. This inflates the apparent efficacy of the interventions evaluated in trials with passive controls, such as the wait-list. It is not known whether and to what extent this is observed in working populations or for more preventive approaches.

Considering the increasing number of interventions being developed, implemented, and potentially evaluated, this review aimed to address the following questions by systematically reviewing the current state of evidence for the efficacy of eHealth interventions in reducing depression, anxiety, and stress in employees (Textbox 1).

**Research questions.**

**Research questions addressed by this review**

- Is there evidence for the improvement in effectiveness over time (ie, are the interventions getting better)?
- What factors, if any, moderate the efficacy?
  Intervention approach; indicated, tailored, tertiary or universal
  Type of control (active vs wait-list)
  Type of intervention: cognitive behavioral therapy, mindfulness, stress management, and other
  Presence of in-person support

## Methods

### Overview

This review complies with the PRISMA (Preferred Reporting Items for Systematic Reviews and Meta-Analyses) guidelines [25]. We aimed to identify all published and unpublished, peer-reviewed, controlled clinical trials of eHealth interventions targeted at employees reporting outcomes on a standardized mental health measure of depression, anxiety, and stress. The systematic review protocol was registered on PROSPERO (International Prospective Register of Systematic Reviews; CRD42020185859).

### Search Strategy

A systematic literature search was conducted using MEDLINE, PsycINFO, Cochrane Register of Controlled Trials, and Embase electronic databases for relevant peer-reviewed articles in English of controlled trials and RCTs published from 2004 (when the first eHealth intervention was identified) [19] to July 2020. Keywords used related to “workplace,” “intervention,” “outcome,” and “study design.” An example of this search strategy is presented in Multimedia Appendix 1. The search terms were developed from our previous systematic review [19]. The tables of contents of the *Journal of Medical Internet Research*, *Journal of Internet Interventions*, *Occupational and Environmental Medicine*, and the *Journal of Occupational and Environmental Medicine*, as well as the reference lists of included studies, were manually searched.

### Study Selection Criteria

#### Eligibility Criteria

The PICOS (Patient/Population, Intervention, Comparison, and Outcomes) framework commonly used to identify components of clinical evidence for systematic reviews in evidence-based medicine, endorsed by the Cochrane Collaboration [26], was used in this review.

##### Participants

Participants had to be in current paid employment and working-age adults aged between 18 and 65 years. Studies were excluded if the sample was defined as volunteer workers, unemployed participants, or general or clinical populations.

##### Interventions

Any eHealth intervention, defined as a therapeutic intervention delivered through a website, smartphone, tablet, or mobile app and designed to improve mental health, was included.

##### Controls

Studies were required to have a control group, defined as either *passive* (care as usual, no contact, or wait-list) or *active* (eg, another eHealth intervention as the comparison group).

##### Outcomes

The study had to report on at least one common mental disorder outcome: depression, anxiety, or stress. All eligible outcomes for each study and domain were included. The outcomes were standardized mean difference (SMD) from preintervention baseline score to immediate postintervention use and the follow-up time point.

The following measurements of common mental disorders were regarded as eligible:

1. Diagnostic interview
2. Self-report diagnosis by a physician, psychologist, or other qualified health professional
3. Self-administered rating scale for mental health (anxiety, depression, or stress)

##### Studies

Studies were excluded from the review if they were not specifically limited to employees and delivered in a workplace setting or used in-person, telephone, and email interventions only.

#### Identification of Studies

After duplicates were removed, 3 independent authors (ES, IC, and MA) screened all titles and abstracts to identify potentially relevant studies. Abstracts and full-text versions of potentially eligible studies were independently assessed by 2 investigators (ES and IC). Eligible studies with individual citations were scanned to ensure that all relevant studies were identified. Disagreements were adjudicated in conjunction with the senior author (NG).

### Data Extraction and Coding

We extracted the mean and SD of standardized measures of stress or distress, depression, and anxiety and the sample size (n) in each arm (intervention and control) at baseline and at each follow-up time point. When sufficient details were not reported, the authors were contacted. Additional data concerning the type of design, intervention and control details, participant characteristics, study duration, length of follow-up, organizational population, and general outcomes were recorded (see the *Results* section). Studies were also coded by intervention type: *universal* interventions targeting relatively healthy participants; *indicated* interventions targeting subclinical symptoms or syndromes; and *tertiary* treatments for explicit diagnoses.

Studies reporting results of the same intervention and sample in different papers (eg, a postintervention and follow-up study) were treated as 1 study, and we used the first follow-up outcome point in the analysis.

We categorized studies according to their mental health outcome measures: anxiety, depression, or stress. Studies with multiple outcomes appear in >1 mental health outcome result.

### Quality Assessment and Risk of Bias Within Studies

The risk of bias in RCTs was assessed using the revised Cochrane risk of bias tool for RCTs (RoB version 2.0) [27], in which 5 domains were independently evaluated by 2 authors: randomization process, deviations from intended interventions, missing outcome data, measurement of the outcome, and selection of the reported result. Each domain was assessed for the risk of bias. Studies were graded as (1) “low risk of bias” when a low risk of bias was determined for all domains, (2) “some concerns” if at least one domain was assessed as raising some concerns but not to be at high risk of bias for any single domain, or (3) “high risk of bias” when a high risk of bias was determined for at least one domain or there were bias concerns in multiple domains [27]. If a study contained >1 intervention arm, each intervention was assessed as 1 study.

### Statistical Analysis

The summary ES was the SMD (calculated as Hedge *g*, with 95% CI) between the intervention and control groups for each outcome measure (anxiety, depression, and stress) [28]. A positive ES (SMD) indicated that the intervention was more efficacious than the control. The level of significance was set at *P*<.05 and 95% CIs. The magnitude of the effect was categorized as large (SMD>0.8), moderate (SMD 0.5-0.8), small (SMD 0.2 to <0.5), or trivial (SMD<0.2) [29]. Pooling of ESs across studies was performed using a random-effects model in Comprehensive Meta-Analysis (Biostat Inc; version 3). We detected and accommodated outliers in the meta-analysis using the random-effects variance shift model and the likelihood ratio test. As the different types of eHealth interventions and approaches used may result in large heterogeneity [30], between-study heterogeneity was quantified using *τ*^2^ (variance of true effects) and further assessed using the Higgins *I*^2^ statistic, which estimates the proportion of observed variance not because of sampling error. An *I*^2^ value of 75% was considered large, 50% was considered moderate, and 25% was considered low [31].

We estimated the results separately for each mental health outcome measure (anxiety, depression, and stress) at the postintervention and first follow-up time points. Meta-regressions were performed for each mental health outcome to identify whether the intervention approach (eg, universal), type of intervention (eg, CBT), type of control (eg, wait-list), or intervention delivery method (web-based vs smartphone) moderated the observed effect.

To evaluate differences in effectiveness over time, we used only within-group ESs from the intervention arms for anxiety, depression, or stress outcomes. ESs were calculated by dividing the difference between pre- and posttreatment means by the pooled SD of the difference [32]. ANOVA was used to assess whether there were any differences between the mean ESs for each year. For a test for trends, the within‐group ESs with positive signs indicated improvement, and negative signs indicated worsening. This method allowed for the difference in effects over time to be accurately calculated, allowing us to explore whether interventions improved with technology.

To assess small study effects (in cases where at least 10 studies were available for analysis), we used a funnel plot for the overall effects and each subgroup analysis, which compared the outcome effects with their SEs. We used the Egger regression test to examine further asymmetry of the funnel plot [33] with statistical significance based on *P*<.10. In cases where at least 10 studies were available and a small study effect was found, we used a Duval and Tweedie trim and fill analysis to quantify the magnitude of the small study effect [34].

## Results

### Search Results

**Figure 1:**
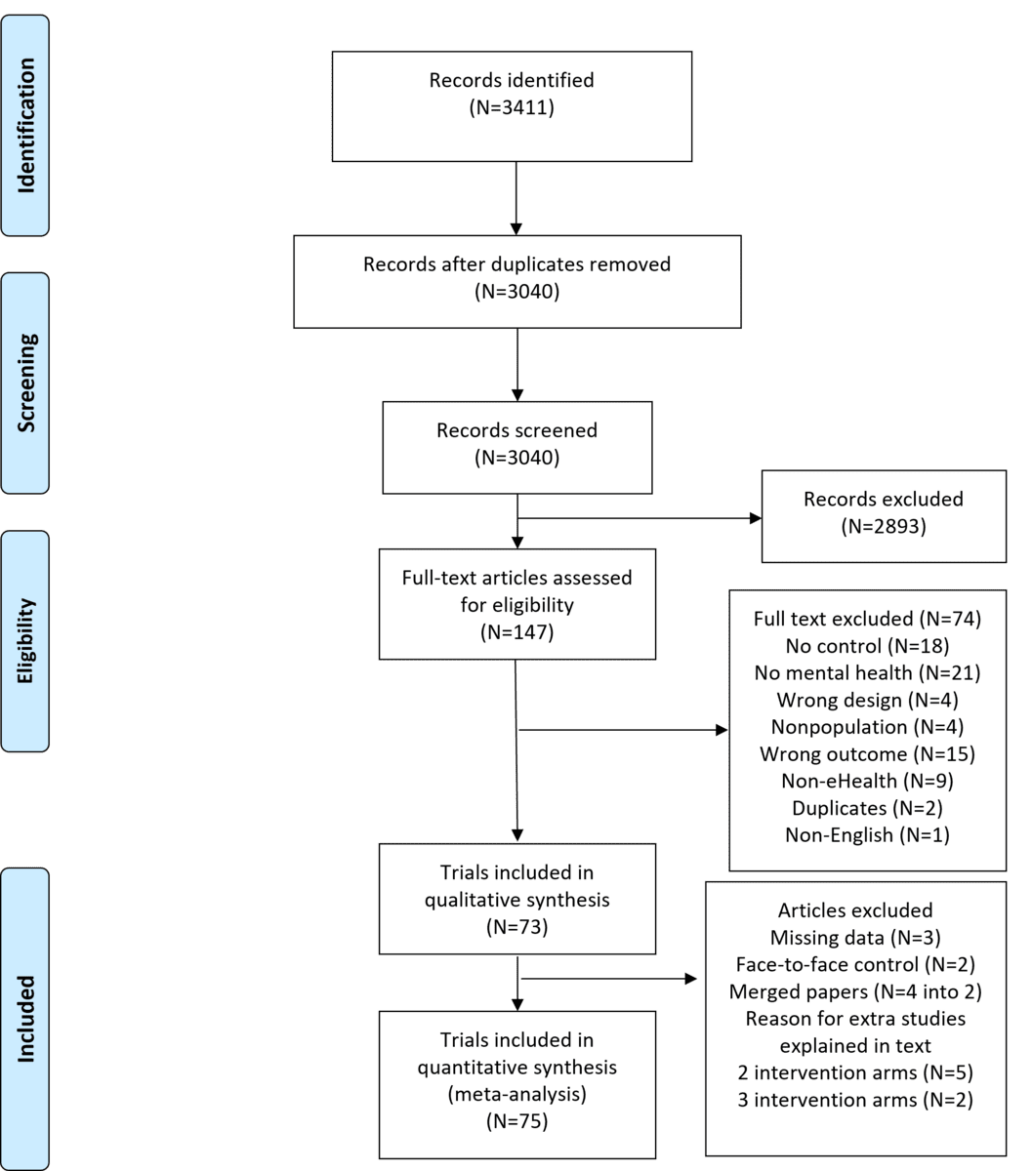
Flow Diagram of included studies.

The search strategy identified 3411 titles (Figure 1). Of the 3411 articles, after the removal of 371 (10.88%) duplicates, 3040 (89.12%) titles and abstracts were reviewed by the authors (ES, IC, and MA). Of these 3040 articles, 2893 (95.16%) articles were excluded based on the eligibility criteria, leaving 147 (4.84%) articles that were potentially relevant to the research question. Their full texts were examined by 2 independent researchers (ES and MA), and discrepancies were decided by the senior author (NG). Of the 147 articles, 74 (50.3%) were excluded for reasons provided in Figure 1. Of the remaining 73 studies, data were missing for 8 (11%) studies. The authors were contacted, and 5 responded with relevant data [35-39]. A total of 3 authors did not respond or were unable to obtain data; thus, these studies were excluded from the meta-analysis [35,40,41]. Of the 73 studies, 4 (5%) articles reported on the same study at different follow-up time points and were merged [42-45], leaving 68 (93%) studies, of which 2 (3%) were excluded as they used face-to-face treatment for control groups [46,47]. The remaining 97% (66/68) of articles were identified as meeting the criteria for quality assessment. Of the 66 studies, 5 (8%) used 2 intervention arms [48-52], and 2 (3%) studies used 3 intervention arms [44,53]. In this case, the intervention arms were treated as individual trials reported as author names; year of publication; and the letters a, b, or c (eg, Smith [a]). The number of participants in the control group was split evenly as a comparative arm to ensure that participants were not counted twice. This process resulted in 75 trials (Figure 1) for the meta-analysis. Multimedia Appendix 2 [36-39,42-45,49-53] provides the list of references.

### Risk of Bias

The individual studies included in the meta-analysis showed that, overall, 12% (9/75) had a low risk of bias, 3% (2/75) had ≥1 concern, and 85% (64/75) had a high risk of bias. Most of the bias was observed in deviations from the intended interventions and missing outcome data, which is not uncommon and is an area for improvement in the eHealth field. An important aspect common to behavioral interventions is that 55% (41/75) of the studies were rated as having a high risk of bias because of the use of a wait-list control group. As such, it was impossible for users to remain blinded (Multimedia Appendix 3 [36-39,42-45,49-53]).

All the included trials used web-based self-report measures to collect the baseline and follow-up data. Multimedia Appendix 4 provides an overview of the scales used and their prevalence.

### Meta-analysis Results

#### Overall Summary of the Identified Interventions

**Figure 2:**
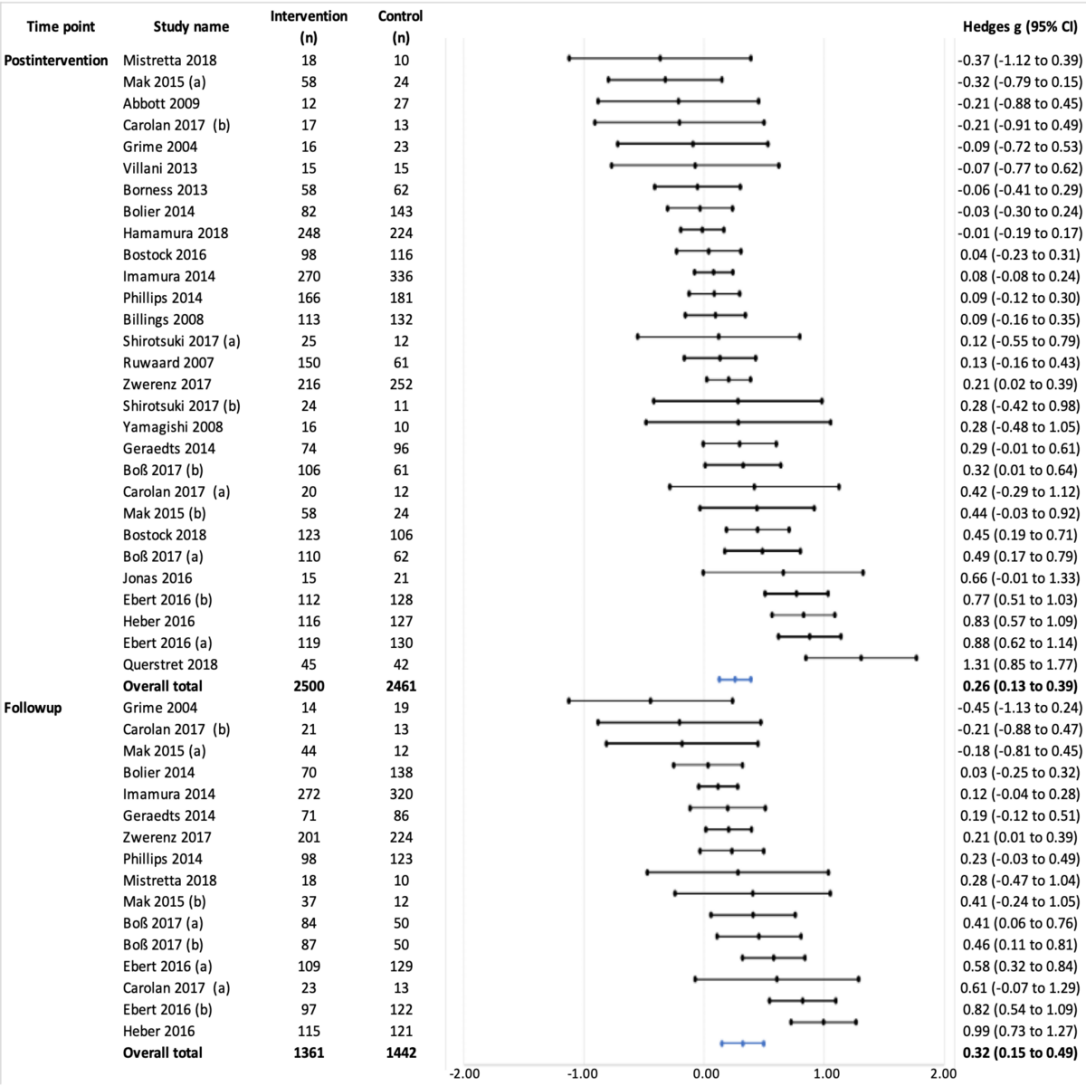
Effects on anxiety symptoms.

The 75 trials reported postintervention data for 14,747 participants. Most trials used universal approaches (47/75, 63%), with fewer indicated (10/75, 13%), tertiary (11/75, 15%), or tailored (7/75, 9%) intervention approaches. Most interventions were delivered via web-based platforms as opposed to smartphone apps. Most of the trials used a wait-list control group. The 3 most common types of interventions described by the authors were CBT, mindfulness, and stress management. The studies were conducted in 15 countries: the United States (16/75, 21%), Japan (15/75, 20%), Germany (13/75, 17%), the United Kingdom (7/75, 9%), the Netherlands (5/75, 7%), Australia (4/75, 5%), Sweden (4/75, 5%), China (2/75, 3%), Europe combined (2/75, 3%), Italy (2/75, 3%), Brazil (1/75, 1%), Finland (1/75, 1%), Hong Kong (1/75, 1%), Singapore (1/75, 1%), and the United States and Canada combined (1/75, 1%). The most common types of participants were health care professionals (18/75, 24%). Interventions for the insurance industry (7/75, 9%), managers (6/75, 8%), information technology (6/75, 8%), male-dominated industries (5/75, 7%), telecommunications (5/75, 7%), schools (3/75, 4%), universities (3/75, 4%), marketing and sales (3/75, 4%), banking (1/75, 1%), and human resources (1/75, 1%) were also evaluated (Figure 2). The term *general employee* is used in Multimedia Appendix 2 when the study did not mention the organization type. A total of 15 trials were conducted across several industries, and only 2 were conducted in employees on sick leave. Further summarized and detailed descriptions of the studies are presented in Table 1 and Multimedia Appendix 5 [36-39,42-45,49-53].

**Table 1:** Summarized description of the selected studies.

Study	Type	Country and population (sample size)	Intervention and duration (sample size)	Control (sample size)	Type of intervention
Grime^a^ [54]	CBT^b^	United Kingdom; Occupational health department (48)	“Beating the Blues” web-based program for depression and anxiety 8 web-based sessions, which last approximately an hour per week (24)	WLC^c^ (24)	Tertiary
Hasson et al^a,d^ [55]	CBT	Sweden; IT and media companies (317)	12-month open access Web-based self-help exercises (129)	WLC+information (174)	Universal
Shimazu et al^a^ [56]	CBT	Japan; construction machinery company (225)	1-month web-based psychoeducation based on social cognitive theory Self-based program with 3 phases (5 chapters; 112)	WLC (113)	Universal
Cook et al^a^ [57]	Stress management	United States; human resource employees (419)	Web-based multimedia health promotion program “Health Connection” (209)	WLC+paper-based information(210)	Universal
Ruwaard et al^a,d^ [58]	CBT	The Netherlands; general employees (239)	7-week web-based program Supported by trained therapists with 10 personalized feedback sessions 1 module per week: awareness, relaxation, worrying, positive self-verbalization, positive assertiveness, and time management In-person and audio training (177)	WLC (62)	Tailored
Billings et al^e^ [59]	CBT	United States; technology company (309)	3-month open-access web-based program (154)	WLC (155)	Tailored
Suzuki et al^a^ [60]	CBT	Japan; university staff (43)	A 2-week program with 4 modules Daily monitoring and feedback and sleep diary Emailed weekly summary and advice (21)	WLC (22)	Universal
Yamagishi et al^a^ [61]	Assertion training	Japan; shift working nurses (60)	9 weeks of 60-minute web-based training provided weekly (30)	WLC (30)	Universal
Abbott et al^a^ [62]	CBT	Australia; industrial organization (53)	Web-based program 7 core modules (26)	WLC (27)	Universal
Bennett et al^a^ [63]	Behavior change	United States; general managers (145)	Web-based open-access ExecuPrev At least 10 hours over 6 months (72)	WLC (73)	Universal
Glück and Maercker^a^ [64]	Mindfulness	Austria, Germany, and Switzerland; universities, car dealerships, broadcasting stations, and health care consulting (50)	Web-based program for 13 days 2 modules, with each module lasting for 6 days with 20 minutes per day (28)	WLC (21)	Universal
Borness et al^a^ [65]	Cognitive training	Australia; public sector general employees (135)	16 weeks of web-based cognitive training based on memory, attention, language, and executive function The program was called “Spark!” with three 20-minute sessions per week (67)	Active control program (68); general knowledge information	Universal
Feicht et al^a^ [66]	Positive psychology	Germany; insurance company (147)	7-week web-based happiness training Weekly modules that took 10 to 15 minutes Emailed instructions once weekly (85)	WLC (62)	Universal
Ketelaar et al [38]^a^	Health Surveillance Model	The Netherlands; nurses and allied health professionals (367)	Tailored, assessed by screening Psyfit: well-being based; healthy participants Strong at work: stress management; learning skills to cope with work stress Color your life: depressive symptoms Don’t Panic Online: panic symptoms for subclinical and mild cases of panic disorder Drinking less: reducing risky alcohol drinking behavior (178)	WLC (188)	Tailored
Lappalainen et al^a,d^ [67]	CBT and ACT^f^	Finland; men aged 28 to 58 years with depression (23)	“P4Well”—a 3-month program, including 3 group meetings, an internet or web portal, mobile phone apps, and personal monitoring devices (11)	WLC (12)	Tertiary
Villani et al^a,d^ [68]	Stress management	Italy; female oncology nurses with high stress (30)	4-week program, with eight 5-minute video clips twice weekly, with a narrative After work on the study phone (15)	Active control with 8 video clips (15)	Indicated
Bolier et al^a,d^ [69]	Health Surveillance Model	The Netherlands; nurses, allied health professionals, and general employees (423)	Tailored, assessed by screening Psyfit: well-being based; healthy participants Strong at work: stress management; learning skills to cope with work stress Color your life: depressive symptoms Don’t Panic Online: panic symptoms for subclinical and mild cases of panic disorder Drinking less: reducing risky alcohol drinking behavior (212)	WLC (211)	Tailored
Deitz et al^a^ [70]	Behavior change	United States; hospital employees with cardiovascular risk (210)	6-week web-based program with weekly modules On the basis of increasing knowledge and reducing risk (105)	WLC (105)	Universal
Ebert et al^a,d^ [10]	Problem-solving training	Germany; teachers (150)	6 weeks, 5 lessons Web-based program with 1 lesson per week and practice between each lesson (75)	WLC (75)	Tertiary
Geraedts et al [42,43] (2 papers)^a,d^	Problem-solving training and cognitive therapy	The Netherlands; banking (231)	Web-based Happy@Work program 6 weekly sessions, participants had to complete an assessment each week to move on (116)	WLC (115)	Tertiary
Imamura et al [44,45] (2 papers)^a,d^	CBT	Japan; IT (762)	6-week web-based program 6 lessons, 1 lesson per week, approximately 30 minutes each Each lesson had homework (381)	WLC+information (381)	Tertiary
Ly et al^a,d^ [71]	ACT	Sweden; middle managers in the private sector (73)	6-week smartphone app 6 modules, 1 per week Audio lecture, text, and exercises supported by student psychologist (36)	WLC (37)	Universal
Mori et al^a,d^ [72]	CBT	Japan; IT engineers with high computer literacy (168)	A 4-week program with homework Web-based 150-minute group class; web-based entries to log daily stresses (85)	WLC (83)	Universal
Phillips et al^a^ [73]	CBT	United Kingdom; transport, health, and communication sectors (637)	5-week web-based “MoodGYM” program 5 modules of 1 hour for preventing and coping with depression (318)	WLC+information (319)	Tertiary
Umanodan et al^a^ [74]	Stress management	Japan; manufacturing company (266)	6-week web-based program 6 lessons (1 per week); self-paced 2-phased learning process (142)	WLC (121)	Universal
Carissoli et al^e^ [75]	Mindfulness	Italy; general employees (56)	3-week smartphone app Practice 2 meditations per day, lasting 15 minutes each (20)	Music; 2 songs per day, lasting approximately 15 minutes each, while doing nothing else (18)	Universal
Cook et al^a^ [76]	Stress management	United States; older IT employees (278)	“HealthyPast50,” a web-based open-access program 3 months, 5 modules (138)	WLC (140)	Universal
Ebert et al^a^ [77]	Behavior change	Germany; teachers (128)	6-week internet-based weekly sessions (64)	WLC (64)	Universal
Guille et al [36]^a^	CBT	United States; medical interns (199)	MoodGYM program, comprising 4 weekly, web-based sessions lasting approximately 30 minutes each (100)	4 weekly mental health information emails (99)	Universal
Mak et al [51]^a^	MBSR^g^	China; university (321)	2-arm intervention: 8-week web-based mindfulness training, 1 lesson per week that took 23 to 30 minutes (107) The second group had the identical training plus health action process approach(107)	WLC (107)	Universal
Prasek^a^ [78]	Mindfulness	Unite States; university (192)	7-week self-guided, web-based mindfulness program called Sherman Project (101)	WLC (91)	Universal
Stansfeld et al^a,d^ [79]	Stress management	United Kingdom; NHS^h^ Mental Health Trust (275)	Web-based team-based health promotion program 6 fortnightly modules for 3 months (216)	WLC (59)	Universal
Volker et al [39]^a^	CBT and PST^i^	The Netherlands; sick-listed (773)	Return@Work, a web-based program with 5 modules Up to 17 sessions, ranging from 6 to 17 (131)	WLC (89)	Tertiary
Yuan^a^ [80]	PST	Hong Kong	Happy@Work, a web-based program with 4 modules (162)	WLC (159)	Universal
Allexandre et al [53]^a^	Mindfulness	United States; general employees from a corporate call center (91)	8-week web-based program with 1 session per week; audio guided; daily articles available and 2 email reminders sent (54) Access to the above plus meeting in groups for 1 hour once a week; group session deep breathing exercise for 2 minutes, 10-minute audio recording, 20- to 30-minute guided meditation, and 20 minutes of discussion questions (37) Same as above plus weeks 3, 6, and 8 were facilitated by a licensed clinical (33)	WLC (37)	Universal
Birney et al^a^ [81]	CBT	United States (300)	6-week CBT mobile phone app “MoodHacker” Brief daily interactions (150)	WLC (150)	Tertiary
Bostock et al^a^ [82]	CBT	United States; office based (270)	8-week web-based Sleepio.com intervention Animated virtual therapist (“The Prof”), sleep diary data, email or SMS text message prompts, and moderated web-based community (135)	WLC (135)	Universal
Dyrbye et al^a^ [83]	Positive psychology	United States; practicing physicians (290)	6-week web-based program One 5-minute domain per week (145)	WLC (145)	Universal
Ebert et al^a,d^ [84] (a)	Stress management	Germany (264)	7-week web-based intervention GET.ON Stress Weekly modules, 45 to 60 minutes each Daily stress diaries and e-coach (psychologist; 132)	WLC (132)	Indicated
Ebert et al^a,d^ [85] (b)	Stress management	Germany (264)	7-week web-based intervention GET.ON Stress Weekly modules, 45 to 60 minutes each Daily stress diaries (132)	WLC (132)	Indicated
Heber et al^a,d^ [86]	Stress management	Germany (264)	7 sessions, with 1 to 2 sessions per week; web-based (132)	WLC (132)	Indicated
Hersch et al^a^ [87]	Stress management	United States; nurses and nurse managers in public hospitals (104)	Web-based intervention BREATHE, with open access for 3 months and 7 modules (52)	WLC (52)	Universal
Imamura et al^a^ [44]	CBT	Japan (1236)	4-week internet access to UTSMed, comprising text and illustrations; 90 pages (276) Same as above but for moderate depression (291) Same as above but for high depression (51)	WLC no depression (285); same as above but for moderate depression (290); same as above but for high depression (43)	Tailored
Jonas et al^a,d^ [88]	CBT	Germany; employees with burnout (59)	A 4-week web-based program “Beratung Hilft” (“counselling helps”) Structured and therapist guided Daily stress diary (18)	WLC (21)	Indicated
Beiwinkel et al^a,d^ [89]	CBT	Germany (180)	“HelpID,” a 12-week web-based program with weekly sessions of 30 to 45 minutes Weekly reminder emails (100)	WLC+information (80)	Tertiary
Boß et al [48] (a)^a^ and (b)^a,d^	CBT	Germany (434)	5-week web-based program with 1 module per week (146) Identical to the GET.ON intervention plus additional adherence-focused guidance by e-coaches (trained psychologist; 144)	WLC (144)	Universal
Carolan et al [49]^a,d^	CBT	United Kingdom (84)	WorkGuru, a web-based program with 7 weekly modules (28) Same as above plus weekly web-based, guided discussion group (28)	WLC (28)	Indicated
Shirotsuki et al [52]^a^	CBT	Japan; office workers in hospitality and sales company (87)	6-week web-based program where participants watched weekly e-learning movie segments (5-10 minutes long); recorded daily mood and weekly homework (29) Same as above plus participants consumed 1 bottle of the supplement soft drink (29)	Recorded their mood state every day on a weekly monitoring sheet (homework; 29)	Universal
Zhang et al^a^ [90]	Awareness training	Singapore; health care workers (80)	4-week smartphone-based program 20-minute briefing session and a daily SMS text messaging service (40)	Information from the Health Promotion Board (40)	Universal
Zwerenz et al^a,d^ [91]	Psychoeducation	Germany; sick-listed and in inpatient rehabilitation (652)	12-week web-based program with weekly reflective 45-minute blogs instructed by a therapist Reminder emails, individualized feedback, audio-guided stress management, homework, and forum (303)	Active control; regular email reminders to use selected information posted on the web about stress management and coping (329)	Tertiary
Bostock et al^a^ [92]	Mindfulness	United Kingdom; pharmaceutical and high-tech employees (238)	8-week Headspace smartphone app 45 days of daily 10- to 20-minute mindfulness meditation (128)	NHS web-based advice for work stress (110)	Universal
Eriksson et al^a^ [93]	Mindful self-compassion program	Sweden; practicing psychologists (101)	6 weeks web-based program Videos with guided instructions (51)	WLC (49)	Universal
Gollwitzer et al [50]^a^	Mental contrasting	Germany; general nurses (129)	Web-based 3-week program; daily written reflections on “what if”; identifying obstacles in the way (41) Same as above plus structured daily time for use (41)	WLC (47)	Universal
Hamamura et al^e^ [94]	CBT	Japan; marketing company (557)	A 4-week smartphone app called “jibun kiroku” Daily activities on an hourly basis Evaluate the quality of their sleep, mood, and energy level (306)	WLC (251)	Indicated
Imamura et al [37]^a,d^	CBT	Japan; telecommunications (706)	6-week 6-lesson web-based program with 30-minute lessons per week Voluntary homework and reminder emails (353)	WLC (353)	Universal
Lilly et al^a^ [95]	MBSR	United States and Canada; emergency telecommunication (323)	7-week web-based (Destress 9-1-1) program, with 1 module per week for 30 minutes; introduction video, texts, and a moderated discussion board; outside practice for up to 45 minutes of daily mindfulness homework Guided audio (163)	WLC (160)	Universal
Mistretta et al^a^ [96]	MBRT^j^	United States; hospital employees (38)	6-week smartphone app (23)	WLC (15)	Indicated
Oishi et al^a^ [97]	CBT	Japan; teachers (240)	12-week web-based “Mind Skill Up Training” program 7 modules plus 1 group session, including homework, mood tracking, audio, and visual narrator, with 6 reminder emails (120)	WLC (120)	Universal
Persson Asplund et al^a,d^ [98]	Stress management	Sweden; middle managers in health care, education, IT, or communications sectors (117)	8 weekly modules 2 to 3 hours per week to complete Personalized written feedback via email from a coach (psychologist; 59)	Active control; weekly mail contact, homework, and access to a moderated discussion forum with other users (58)	Indicated
Querstret et al^a^ [99]	MBCT^k^	United Kingdom (118)	4-week web-based program (60)	WLC (58)	Universal
Song et al^a^ [100]	Health Surveillance Model	Japan (1526)	16-week smartphone app “Karada-no-kimochi” Records weekly and daily moods (612)	WLC (914)	Universal
Coelhoso et al^a,d^ [101]	Mindfulness meditation	Brazil; female private hospital employees (490)	8-week smartphone app 4 with classes per week Each class contained a brief theoretical portion and a 15-minute guided practice Participants wrote reflections in a gratitude journal for 20 minutes per week Pop-ups, notifications, and feedback scores (250)	Active control; similar program (240)	Universal
Stratton et al^a^ [102]	Disclosure decision aid tool	Australia (107)	READY web-based disclosure decision aid tool 2-week access; 7 modules, requiring approximately 60 minutes to complete (53)	Information provided about disclosure on leading NGO^l^ website (54)	Tertiary
Weber et al^a^ [103]	Behavior change	Germany, England, and Northern Ireland (532)	4-week smartphone app “Kelaa Mental Resilience App” 28 sessions daily with 2 key modules Tracks mood and health and provides feedback; 6 to 7 daily sessions, each approximately 2 to 4 minutes (210)	WLC (322)	Universal
Deady et al^a^ [104]	Behavior activation and mindfulness	Australia; male-dominated industries (2257)	HeadGear, a smartphone app 30-day “challenge” daily (5-10 minutes per day) Risk calculator with personalized feedback, mood tracker, a toolbox of skills, and support service helplines (1131)	Active control same intervention but without risk calculator and mood tracker (1144)	Universal

^a^Randomized controlled trial.

^b^CBT: cognitive behavioral therapy.

^c^WLC: wait-list control.

^d^Guided intervention.

^e^Controlled trial.

^f^ACT: acceptance and commitment therapy.

^g^MBSR: mindfulness-based stress reduction.

^h^NHS: National Health Service.

^i^PST: problem-solving therapy.

^j^MBRT: mindfulness-based resilience training.

^k^MBCT: mindfulness-based cognitive therapy.

^l^NGO: nongovernmental organization.

#### Efficacy for Anxiety

**Figure 3:**
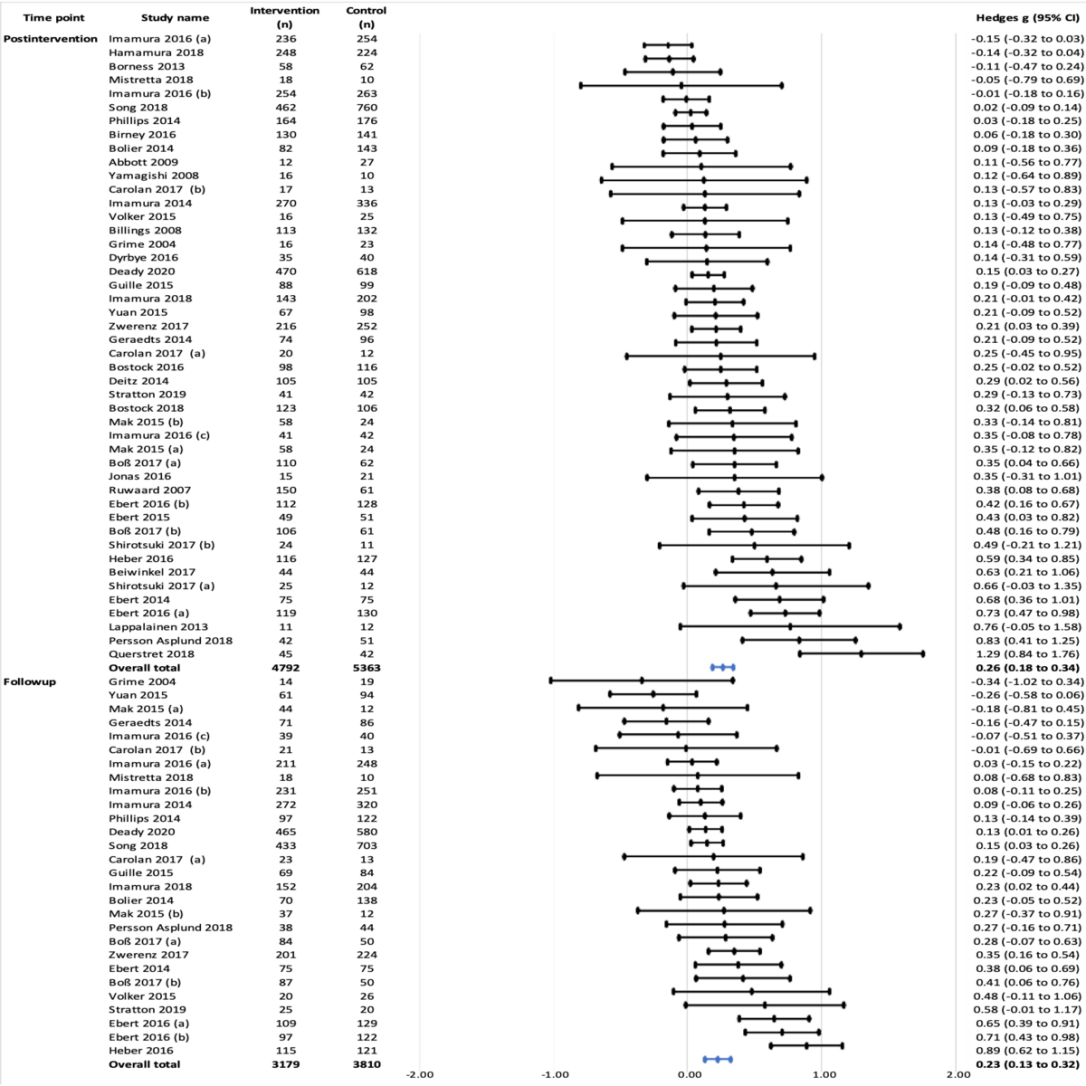
Effects on anxiety symptoms at postintervention and follow-up time points.

Overall, the 29 trials that measured anxiety outcomes showed a significant, small positive effect at the postintervention time point (Hedges *g*=0.26, 95% CI 0.13-0.39; *P*<.001), with high heterogeneity (*I*^2^=77.49%; *τ*^2^=0.08). Approximately 21% (16/75) of studies reported follow-up outcomes, showing a small positive effect (Hedges *g*=0.32, 95% CI 0.15-0.50; *P*<.001), with similarly large heterogeneity (*I*^2^=76.55%; *τ*^2^=0.08; Figure 3).

#### Efficacy for Depression

An overall small positive effect on depression at the postintervention time point (Hedges *g*=0.26, 95% CI 0.19-0.34; *P*<.001) was estimated by combining 46 trials. Moderate heterogeneity was observed (*I*^2^=66.96%; *τ*^2^=0.04). At the postintervention time point, one outlier was detected. By removing this outlier with a very large ES, a statistically significant but small magnitude positive effect remained (Hedges *g*=0.24, 95% CI 0.17-0.32; *P*<.001). Approximately 60% (45/75) of the studies reported follow-up outcomes. The follow-up effects on depression in 37% (28/75) of studies were very similar (Hedges *g*=0.23, 95% CI 0.13-0.32; *P*<.001), again with moderate heterogeneity (*I*^2^=68.27%; *τ*^2^=0.03; Figure 2).

#### Efficacy for Stress

**Figure 4:**
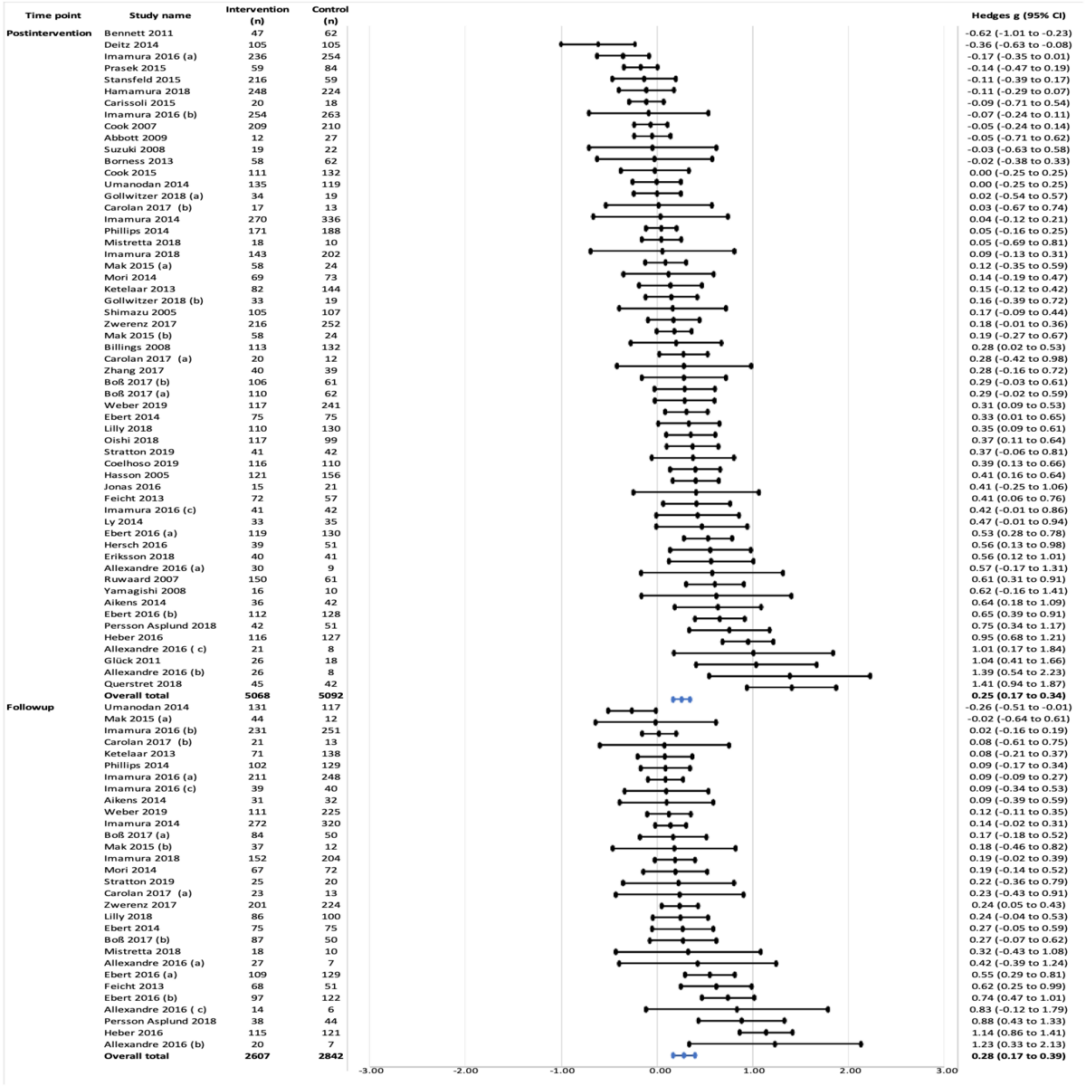
Effects on stress symptoms at postintervention and follow-up time points.

Stress was the most common mental health outcome assessed in this study. In 76% (57/75) of studies, a small positive effect was found overall at the postintervention time point (Hedges *g*=0.25, 95% CI 0.17-0.34; *P*<.001). However, large heterogeneity was detected (*I*^2^=76.11%; *τ*^2^=0.07). Overall, a significant but small positive effect was observed at follow-up in 40% (30/75) of studies (Hedges *g*=0.28, 95% CI 0.17-0.40; *P*<.001), with moderate heterogeneity detected (*I*^2^=73.36%; *τ*^2^=0.06; Figure 4).

#### Moderating Factors for Outcome Efficacy

##### Anxiety

Mixed-effects meta-regression (Table 2) showed that for anxiety, the difference between approach efficacy was statistically significant (*Q*=21.72; *P*<.001; *R*^2^=0.64). Stress management interventions (Hedges *g*=0.79, 95% CI 0.64-0.93; *P*<.001) and mindfulness (Hedges *g*=0.42, 95% CI 0.14-0.60; *P*<.001) interventions were more effective than CBT (Hedges *g*=0.11, 95% CI 0.04-0.19; *P*=.004) and *other* interventions (eg, cognitive training; Hedges *g*=0.13, 95% CI 0.01-0.25; *P*=.04). There were no significant between-group differences between the intervention approach, level of support, or type of control used.

**Table 2:** Meta-regression analysis for moderators of true effect on each mental health outcome at the postintervention time point.

Intervention component and study design factor				Study, n (%)		Meta-analysis				Heterogeneity							Meta-regression between-group tests				
						Hedges *g* (95% CI)		*P* value		*Q*		*P* value		*I* ^2^		*Q*			*P* value		*R* ^2^
**Anxiety**																					
	**Type of intervention**																				
		CBT^a^	14 (19)		0.11 (0.04 to 0.19)		.004		14.41		.35		9.78		21.72			<.001		0.64	
		Mindfulness	5 (7)		0.42 (0.14 to 0.6)		<.001		27.99		<.001		85.71		21.72			<.001		0.64	
		Stress management	4 (5)		0.79 (0.64 to 0.93)		<.001		6.47		.09		53.6		21.72			<.001		0.64	
		Other	6 (8)		0.13 (0.01 to 0.25)		.04		5.45		.35		8.2		21.72			<.001		0.64	
	**Intervention approach**																				
		Indicated	9 (12)		0.44 (0.33 to 0.54)		<.001		60.13		<.001		86.70		3.48			.32		0.03	
		Tailored	3 (4)		0.06 (−0.09 to 0.22)		.44		0.76		.68		0.000		3.48			.32		0.03	
		Tertiary	5 (7)		0.14 (0.04 to 0.23)		.006		2.85		.58		0.000		3.48			.32		0.03	
		Universal	12 (16)		0.29 (0.17 to 0.4)		<.001		38.03		<.001		71.07		3.48			.32		0.03	
	**Person support**																				
		Yes	14 (19)		0.32 (0.24 to 0.4)		<.001		68.96		<.001		81.15		1.02			.31		0.01	
		No	15 (20)		0.16 (0.07 to 0.24)		<.001		47.33		<.001		70.42		1.02			.31		0.01	
	**Type of control**																				
		Active	4 (5)		0.26 (0.07 to 0.45)		.009		5.14		.16		41.64		0.08			.78		0	
		WLC^b^	25 (33)		0.25 (0.19 to 0.31)		<.001		119.23		<.001		79.87		0.08			.78		0	
**Depression**																					
	**Type of intervention**																				
		CBT	23 (31)		0.11 (0.06 to 0.17)		<.001		44.12		.003		50.13		20.32			<.001		0.47	
		Mindfulness	5 (7)		0.46 (0.28 to 0.64)		<.001		16.14		.003		75.22		20.32			<.001		0.47	
		Stress management	4 (5)		0.61 (0.47 to 0.75)		<.001		4.03		.26		25.52		20.32			<.001		0.47	
		Other	14 (19)		0.15 (0.09 to 0.21)		<.001		21.25		.07		38.83		20.32			<.001		0.47	
	**Intervention approach**																				
		Indicated	9 (12)		0.32 (0.21 to 0.42)		<.001		45.95		<.001		85.59		4.45			.22		0.01	
		Tailored	6 (8)		0.04 (−0.06 to 0.14)		.41		12.13		.03		58.79		4.45			.22		0.01	
		Tertiary	11 (15)		0.2 (0.12 to 0.28)		<.001		18.98		.04		47.31		4.45			.22		0.01	
		Universal	20 (27)		0.19 (0.13 to 0.25)		<.001		44.01		.001		56.83		4.45			.22		0.01	
	**Person support**																				
		Yes	20 (27)		0.33 (0.27 to 0.4)		<.001		42.04		.002		54.8		7.20			.007		0.27	
		No	26 (35)		0.11 (0.06 to 0.16)		<.001		65.58		<.001		61.88		7.20			.007		0.27	
	**Type of control**																				
		Active	9 (12)		0.19 (0.11 to 0.28)		<.001		16.78		.03		52.33		0.00			.95		0.00	
		WLC	37 (49)		0.18 (0.14 to 0.23)		<.001		119.35		<.001		69.84		0.00			.95		0.00	
**Stress**																					
	**Type of intervention**																				
		CBT	19 (25)		0.1 (0.04 to 0.16)		.001		47.89		<.001		62.42		10.12			.02		0.09	
		Mindfulness	15 (20)		0.42 (0.31 to 0.53)		<.001		46.31		<.001		69.77		10.12			.02		0.09	
		Stress management	14 (19)		0.28 (0.2 to 0.37)		.006		71.38		<.001		88.79		10.12			.02		0.09	
		Other	9 (12)		0.12 (0.04 to 0.21)		<.001		37.74		<.001		65.56		10.12			.02		0.09	
	**Intervention approach**																				
		Indicated	9 (12)		0.38 (0.28 to 0.49)		<.001		56.76		<.001		85.91		3.26			.35		0.00	
		Tailored	6 (8)		0.07 (−0.03 to 0.16)		.16		27.10		<.001		81.55		3.26			.35		0.00	
		Tertiary	5 (7)		0.12 (0.09 to 0.22)		.01		4.78		.31		16.3		3.26			.35		0.00	
		Universal	37 (49)		0.18 (0.13 to 0.24)		<.001		125.06		<.001		71.21		3.26			.35		0.00	
	**Person support**																				
		Yes	22 (29)		0.33 (0.26 to 0.39)		<.001		80.20		<.001		73.82		6.79			.009		0.15	
		No	35 (47)		0.09 (0.04 to 0.14)		.001		121.76		<.001		72.08		6.79			.009		0.15	
	**Type of control**																				
		Active	7 (9)		0.17 (0.05 to 0.29)		.006		18.38		.005		67.36		0.03			.87		0.00	
		WLC	50 (67)		0.18 (0.14 to 0.23)		<.001		215.97		<.001		77.31		0.03			.87		0.00	

^a^CBT: cognitive behavioral therapy.

^b^WLC: wait-list control.

##### Depression

Similarly, significant differences were observed in the ESs for depression across the types of interventions (*Q*=21.72; *P*<.001; *R*^2^=0.64). Again, stress management interventions (Hedges *g*=0.61, 95% CI 0.47-0.75; *P*<.001) and mindfulness (Hedges *g*=0.46, 95% CI 0.28-0.64; *P*<.001) interventions were more effective than CBT (Hedges *g*=0.11, 95% CI 0.06-0.17; *P*<.001) and *other* interventions (Hedges *g*=0.15, 95% CI 0.09-0.21; *P*<.001). Supported interventions had a higher ES (*g*=0.33, 95% CI 0.27-0.40; *P*<.001) than unsupported (Hedges *g*=0.11, 95% CI 0.06-0.16; *P*<.001) interventions (difference *Q*=7.20; *P*=.007; *R*^2^=0.27). There were no significant differences between the intervention approach and the type of control used.

##### Stress

Differences were observed in the ESs across the type of intervention (*Q*=10.12; *P*=.02; *R*^2^=0.09). Mindfulness interventions had the largest ES (Hedges *g*=0.42, 95% CI 0.31-0.53; *P*<.001), followed by stress management (Hedges *g*=0.28, 95% CI 0.20-0.37; *P*=.006), which was more effective than CBT (Hedges *g*=0.10, 95% CI 0.04-0.16; *P*=.001) and *other* interventions (Hedges *g*=0.12, 95% CI 0.04-0.21; *P*<.001). Supported interventions had a higher ES (Hedges *g*=0.33, 95% CI 0.26-0.39; *P*<.001) than unsupported (Hedges *g*=0.09, 95% CI 0.04-0.14; *P*=.001). The difference between these factors was statistically significant (*Q*=6.79; *P*=.009; *R*^2^=0.15). For stress, there were no significant differences between the intervention approach and type of control used.

#### Effectiveness of eHealth Interventions Over Time

##### Overview

**Figure 5:**
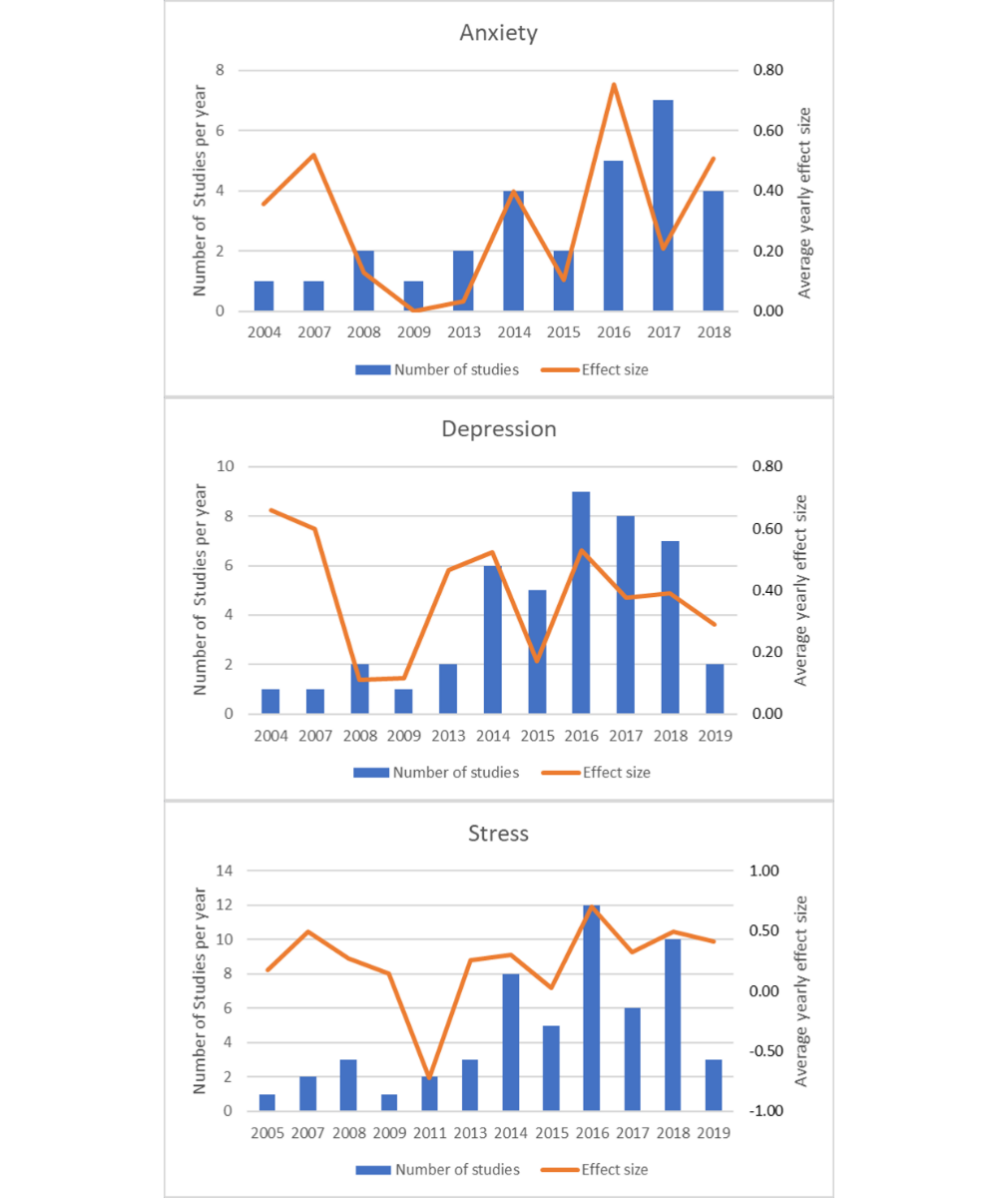
Mean within-intervention group effect sizes reported in trials each year.

Over 5 times more studies evaluating eHealth interventions have been published since 2013 (n=64) than between 2004 and 2013 (n=11). However, the effectiveness of eHealth interventions did not seem to improve over time. The mean within-group ES reported in each year of the eHealth intervention arms in trials published since 2004 remained unchanged, with no significant SMD observed over time for anxiety (*F*_1,9_=0.28; *P*=.61), depression (*F*_1,10_=0.31; *P*=.59), or stress (*F*_1,11_=0.75l; *P*=.41; Figure 5). There appears to have been a nadir in the effectiveness of studies published immediately after the financial crash and recession of 2008/2009.

##### Small Study Effect

A funnel plot for each mental health outcome is shown in Multimedia Appendix 6. No significant asymmetry was found for anxiety outcomes (n=29; Egger intercept 0.24; *P*=.80). However, significant asymmetry was observed for depression (n=46; Egger intercept 1.71; *P*=.001). After conducting a trim and fill analysis, 3% (2/75) of studies were imputed; the observed postintervention ES was adjusted to Hedges *g*=0.28 (95% CI 0.18-0.39). Similarly, significant asymmetry was observed in stress (n=57; Egger intercept 1.89; *P*=.004). After conducting a trim and fill analysis, 23% (17/75) of studies were imputed; the observed postintervention ES was adjusted to Hedges *g*=0.08 (95% CI 0.00-0.19), suggesting a greater effect reported in smaller studies.

## Discussion

### Principal Findings

This updated review aimed to synthesize the burgeoning literature on the efficacy of eHealth interventions for anxiety, depression, and stress outcomes in organizational settings and employee samples. We identified 52 new trial interventions published since the 23 identified in our prior review in 2017 [19], a tripling of the body of evidence. The systematic search identified 75 relevant trials for the meta-analysis, delivering eHealth interventions either on the web or via a smartphone, with a combined sample of 14,747 employees.

eHealth interventions reduced mental health symptoms immediately after use, with small positive effects observed in anxiety (Hedges *g*=0.26), depression (Hedges *g*=0.26), and stress (Hedges *g*=0.25), and data from trials with longer follow-up periods showed similar effects. These results are comparable with those of previous reviews, where small overall effects were found at the postintervention and follow-up time points [19,20]. These results imply that since 2017, the efficacy of eHealth interventions compared with control conditions reported in trials has remained unchanged, suggesting that the effectiveness of eHealth interventions does not seem to be improving over time. An analysis of the within-group effect observed in the intervention arms confirmed that since 2004, there has been no apparent systematic improvement in the effectiveness of these interventions. This is a surprising finding, given the enormous literature on methods to improve engagement with such interventions, greater penetration of technology to populations that do not access health care, and the increasing number of interventions delivered as apps, which reportedly improve access. All of these are commonly cited as factors favoring eHealth as a mode of intervention delivery or improving effectiveness.

The small but significant effect at follow-up suggests that eHealth interventions might have sustained positive effects on mental health. However, only half of the studies assessed follow-up outcomes, which may reflect a reporting bias. Supporting this, null, and in some cases, even negative, effects on mental health were observed in 27% (20/75) of the studies, and a greater proportion—60% (45/75)—did not report follow-up outcomes.

Less than one-fifth of all the trials used active controls. However, we found no significant differences in the efficacy of interventions tested against active versus passive controls overall or for any individual mental health outcome (Table 2). These findings do not confirm the *digital placebo effect*, which has been found in other eHealth reviews in clinical populations, where the ESs were lower in trials that used active controls than in those that used passive controls [24]. Given that most (47/75, 63%) of these trials were not conducted in clinically unwell populations but were delivered universally to employees, this contrary finding may have implications for the types of control recommended for different settings of eHealth trials and for future framework analysis or guidelines.

The primary moderator of efficacy appeared to be the content of the intervention, with stress management and mindfulness-based interventions being seemingly more efficacious than CBT-based interventions. This suggests that CBT, which was adapted from its intended use in a more personalized and clinical setting, may have less useful content, especially for use in organizations with universal delivery, than other approaches adapted for more universal or indicated tools such as mindfulness and stress management.

In our previous review, stronger effects were seen in eHealth interventions that were supported [19], and other studies have found increased effects when eHealth interventions were supported by trained mental health professionals [105]. The positive impacts of supported interventions may suggest that even in generally subclinical populations, a combination of eHealth and adjunctive support is the most effective in reducing mental health symptoms in employees.

Previous research has pointed to the importance of tailoring eHealth interventions to match individual user needs, as mental health symptoms differ from person to person [106]. However, this review did not support the notion that the tailoring of interventions provides any benefit to improving mental health. The limited evidence available suggests no greater efficacy when using tailored interventions than when using interventions delivered universally or for indicated or even unwell samples. In fact, 43% (3/7) of the tailored interventions showed potential for harm in at least one mental health outcome.

### Limitations

This study had some limitations. Significant heterogeneity was detected; however, this is not uncommon in this field or in meta-analytic research [24]. Differences across studies should be considered when interpreting these findings. It must also be acknowledged that some of the follow-up analyses were underpowered and that the findings should be interpreted tentatively. Furthermore, this review did not include any gray literature, and some authors did not provide data. Finally, the individual study pooled data calculated using the Comprehensive Meta-Analysis by Biostat Inc are not presented in this manuscript. Instead, we report the raw data of the pre-post means, SDs, and sample sizes in Table 1.

### Future Directions

As we were unable to detect that eHealth interventions are improving over time, to design effective eHealth interventions, a better understanding of the factors that may influence efficacy is required. This study did not consider engagement and adherence and only considered a small number of potential moderating factors for each intervention. A subsequent framework analysis model is being undertaken to establish the potential beneficial or harmful features of the different types of interventions. This is an important future direction and requires further in-depth analysis as eHealth interventions have the potential to offer a range of novel self-management tools for employees with clinical and subclinical mental health conditions.

Another recommendation is to develop a standard framework for eHealth interventions to best understand the features that have therapeutic benefits and those that may potentially cause harm. This will guide developers to ensure that eHealth interventions are designed in the most effective manner. A framework may address the significant heterogeneity within studies; if the development standards are regulated against this framework, the differences in the features of the interventions and how they are delivered may be reduced. This research suggests that those evaluating interventions or designing protocols should carefully consider the level of support provided when interpreting reported ESs.

### Conclusions

This review and meta-analysis confirms that eHealth interventions have a small but positive impact overall on reducing mental health symptoms in employees. There was significant heterogeneity between trials; however, overall, stress management and mindfulness interventions comprising in-person support appeared to be the most effective. Organizations should carefully consider the interventions delivered within the workplace; otherwise, they may not see long-term value in their return on investments. A substantial minority of intervention trials have demonstrated no efficacy, and a few may even be harmful. There is not enough evidence to make recommendations for the preferential use or development of therapeutically tailored interventions. Disappointingly, we found no evidence that despite the advancements in technology and the enormous resources in time, research, and finance devoted to this area for over a decade, better interventions are being produced. Hopefully, these small ESs do not represent the optimum outcome in organizational settings, and stakeholders and researchers should focus on improving effectiveness and efficacy or comparing and understanding the effects of current interventions. Despite the small effectiveness, this level of reduction could result in a large economic value in an organizational setting.

## Appendix

Multimedia Appendix 1 Example of search terms.

Multimedia Appendix 2 References of the included studies.

Multimedia Appendix 3 Estimated risk of bias.

Multimedia Appendix 4 Mental health measures.

Multimedia Appendix 5 Detailed description of the identified studies and results.

Multimedia Appendix 6 Overall studies small study effect.

## Abbreviations

CBT: cognitive behavioral therapy
ES: effect size
PICOS: Patient/Population, Intervention, Comparison, and Outcomes
PRISMA: Preferred Reporting Items for Systematic Reviews and Meta-Analyses
PROSPERO: International Prospective Register of Systematic Reviews
RCT: randomized controlled trial
SMD: standardized mean difference
