# Supplementary material for: Trends in Effectiveness of Organizational eHealth Interventions in Addressing Employee Mental Health: Systematic Review and Meta-analysis
Source: J Med Internet Res. 2022 Sep 27;24(9):e37776. doi: 10.2196/37776 (PMC9555335; doi:10.2196/37776)
Supplement: Multimedia Appendix 1 [file jmir_v24i9e37776_app1.docx]

**Multimedia Appendix 1: Example of search terms**

| **KEYWORDS - FROM PROJECT PROPOSAL / DESCRIPTION / RESEARCH LITERATURE [Prior Reading]** | | | |
| --- | --- | --- | --- |
| **MEDLINE (via OvidSP) - Medical Subject Headings [MeSH] Thesaurus** | | | |
| **Population** | **Intervention** | **Outcomes** | **Study design** |
| employment.ti. | e-health | stress.tw. | RCT.tw. [Tittle/Abstract] |
| job.ti. | telemedicine.tw. | mental health.ti. | randomised control trial.tw. [Tittle/Abstract] |
| work*.ti. | online.tw. | mental illness.ti | random allocation.tw. |
| worker*.ti. | internet.tw. | mental disorder*.ti. | exp randomized control trial |
| exp employment | internet intervention*.tw. | depress*.tw. | clinical trial.tw |
| work place.tw. | web-based.tw. | anxi*.tw. | controlled clinical trial.tw |
| workplace.tw. | therapy*.tw | affective symptoms.sh. | pre post trial.tw. |
| occupation*.tw. | prevent*.tw. | depression.sh |  |
| work*.tw. | stress management.tw. | anxiety.sh. |  |
| employee.tw. | cognitive behavio?r* therapy.tw | mental disorder*.sh. |  |
| manager*.tw. | CBT.tw | mental health.sh |  |
| Employment[Mesh:NoExp] | smartphone.tw. | stress* [Tittle/Abstract] |  |
| Occupational Groups[Mesh] | intervention*.tw. | mental ill* [tw] |  |
| Professional[Mesh] | app*tw. |  |  |
| Occupations[Mesh] | web based.tw |  |  |
| Workplace[Mesh] | mHealth.tw |  |  |
|  | computeri?ed.tw. |  |  |
|  | digital.tw |  |  |
|  | program*.tw |  |  |
|  | Self Care/ exp |  |  |
|  | Internet/ exp |  |  |
|  | Computerized/ exp |  |  |
|  | Therapy, Computer-Assisted or Computers exp |  |  |

Footnote: all items in the columns are combined by OR, then each overall column was combined by AND, for example Population AND Intervention AND Outcomes AND Study Design
