# Supplementary material for: Trends in Effectiveness of Organizational eHealth Interventions in Addressing Employee Mental Health: Systematic Review and Meta-analysis
Source: J Med Internet Res. 2022 Sep 27;24(9):e37776. doi: 10.2196/37776 (PMC9555335; doi:10.2196/37776)
Supplement: Multimedia Appendix 2 [file jmir_v24i9e37776_app2.docx]

(Grime, 2004, Hasson et al., 2005, Shimazu et al., 2005, Cook et al., 2007, Cook et al., 2015, Deady et al., 2017, Ebert et al., 2015, Geraedts et al., 2014a, Geraedts et al., 2014b, Gluck and Maercker, 2011, Ruwaard et al., 2007, Billings et al., 2008, Suzuki et al., 2008, Yamagishi et al., 2008, Bennett et al., 2011, Borness et al., 2013, Feicht et al., 2013, Ketelaar et al., 2013, Lappalainen et al., 2013, Villani et al., 2013, Deitz et al., 2014, Ebert et al., 2014, Imamura et al., 2014, Imamura et al., 2015, Mori et al., 2014, Phillips et al., 2014, Umanodan et al., 2014, Carissoli et al., 2015, Guille et al., 2015, Mak et al., 2015, Stansfeld et al., 2015, Volker et al., 2015, Allexandre et al., 2016, Birney et al., 2016, Bostock et al., 2016, Ebert et al., 2016a, Ebert et al., 2016b, Heber et al., 2016, Hersch et al., 2016, Imamura et al., 2016, Beiwinkel et al., 2017, Carolan et al., 2017, Jonas et al., 2017, Shirotsuki et al., 2017, Zhang et al., 2017, Boss et al., 2018, Zwerenz et al., 2017, Eriksson et al., 2018, Gollwitzer et al., 2018, Hamamura et al., 2018, Imamura et al., 2018, Mistretta et al., 2018, Oishi et al., 2018, Querstret et al., 2018, Song and Kanaoka, 2018, Coelhoso et al., 2019, Stratton et al., 2019, Weber et al., 2019, Bostock et al., 2019, Abbott et al., 2009, Bolier et al., 2014, Ly et al., 2014, Dyrbye et al., 2016, Lilly et al., 2019, Persson Asplund et al., 2018, Yuan, 2015, Prasek, 2015)

ABBOTT, J.-A., KLEIN, B., HAMILTON, C. & ROSENTHAL, A. 2009. The Impact of Online Resilience Training for Sales Managers on Wellbeing and Work Performance. *E-Journal of Applied Psychology,* 5**,** 89-95.

ALLEXANDRE, D., BERNSTEIN, A. M., WALKER, E., HUNTER, J., ROIZEN, M. F. & MORLEDGE, T. J. 2016. A Web-Based Mindfulness Stress Management Program in a Corporate Call Center: A Randomized Clinical Trial to Evaluate the Added Benefit of Onsite Group Support. *J Occup Environ Med,* 58**,** 254-64.

BEIWINKEL, T., EISSING, T., TELLE, N. T., SIEGMUND-SCHULTZE, E. & ROSSLER, W. 2017. Effectiveness of a Web-Based Intervention in Reducing Depression and Sickness Absence: Randomized Controlled Trial. *J Med Internet Res,* 19**,** e213.

BENNETT, J. B., BROOME, K. M., SCHWAB-PILLEY, A. & GILMORE, P. 2011. A web-based approach to address cardiovascular risks in managers: results of a randomized trial. *J Occup Environ Med,* 53**,** 911-8.

BILLINGS, D. W., COOK, R. F., HENDRICKSON, A. & DOVE, D. C. 2008. A web-based approach to managing stress and mood disorders in the workforce. *J Occup Environ Med,* 50**,** 960-8.

BIRNEY, A. J., GUNN, R., RUSSELL, J. K. & ARY, D. V. 2016. MoodHacker Mobile Web App With Email for Adults to Self-Manage Mild-to-Moderate Depression: Randomized Controlled Trial. *JMIR Mhealth Uhealth,* 4**,** e8.

BOLIER, L., KETELAAR, S. M., NIEUWENHUIJSEN, K., SMEETS, O., GÄRTNER, F. R. & SLUITER, J. K. 2014. Workplace mental health promotion online to enhance well-being of nurses and allied health professionals: A cluster-randomized controlled trial. *Internet Interventions,* 1**,** 196-204.

BORNESS, C., PROUDFOOT, J., CRAWFORD, J. & VALENZUELA, M. 2013. Putting brain training to the test in the workplace: a randomized, blinded, multisite, active-controlled trial. *PLoS One,* 8**,** e59982.

BOSS, L., LEHR, D., SCHAUB, M. P., PAZ CASTRO, R., RIPER, H., BERKING, M. & EBERT, D. D. 2018. Efficacy of a web-based intervention with and without guidance for employees with risky drinking: results of a three-arm randomized controlled trial. *Addiction,* 113**,** 635-646.

BOSTOCK, S., CROSSWELL, A. D., PRATHER, A. A. & STEPTOE, A. 2019. Mindfulness on-the-go: Effects of a mindfulness meditation app on work stress and well-being. *J Occup Health Psychol,* 24**,** 127-138.

BOSTOCK, S., LUIK, A. I. & ESPIE, C. A. 2016. Sleep and Productivity Benefits of Digital Cognitive Behavioral Therapy for Insomnia: A Randomized Controlled Trial Conducted in the Workplace Environment. *J Occup Environ Med,* 58**,** 683-9.

CARISSOLI, C., VILLANI, D. & RIVA, G. 2015. Does a meditation protocol supported by a mobile application help people reduce stress? Suggestions from a controlled pragmatic trial. *Cyberpsychol Behav Soc Netw,* 18**,** 46-53.

CAROLAN, S., HARRIS, P. R., GREENWOOD, K. & CAVANAGH, K. 2017. Increasing engagement with an occupational digital stress management program through the use of an online facilitated discussion group: Results of a pilot randomised controlled trial. *Internet Interv,* 10**,** 1-11.

COELHOSO, C. C., TOBO, P. R., LACERDA, S. S., LIMA, A. H., BARRICHELLO, C. R. C., AMARO, E., JR. & KOZASA, E. H. 2019. A New Mental Health Mobile App for Well-Being and Stress Reduction in Working Women: Randomized Controlled Trial. *J Med Internet Res,* 21**,** e14269.

COOK, R. F., BILLINGS, D. W., HERSCH, R. K., BACK, A. S. & HENDRICKSON, A. 2007. A field test of a web-based workplace health promotion program to improve dietary practices, reduce stress, and increase physical activity: randomized controlled trial. *J Med Internet Res,* 9**,** e17.

COOK, R. F., HERSCH, R. K., SCHLOSSBERG, D. & LEAF, S. L. 2015. A Web-based health promotion program for older workers: randomized controlled trial. *J Med Internet Res,* 17**,** e82.

DEADY, M., CHOI, I., CALVO, R. A., GLOZIER, N., CHRISTENSEN, H. & HARVEY, S. B. 2017. eHealth interventions for the prevention of depression and anxiety in the general population: a systematic review and meta-analysis. *BMC Psychiatry,* 17**,** 310.

DEITZ, D., COOK, R. F., HERSCH, R. K. & LEAF, S. 2014. Heart healthy online: an innovative approach to risk reduction in the workplace. *J Occup Environ Med,* 56**,** 547-53.

DYRBYE, L. N., WEST, C. P., RICHARDS, M. L., ROSS, H. J., SATELE, D. & SHANAFELT, T. D. 2016. A randomized, controlled study of an online intervention to promote job satisfaction and well-being among physicians. *Burnout Research,* 3**,** 69-75.

EBERT, D. D., BERKING, M., THIART, H., RIPER, H., LAFERTON, J. A. C., CUIJPERS, P., SIELAND, B. & LEHR, D. 2015. Restoring depleted resources: Efficacy and mechanisms of change of an internet-based unguided recovery training for better sleep and psychological detachment from work. *Health Psychol,* 34S**,** 1240-1251.

EBERT, D. D., HEBER, E., BERKING, M., RIPER, H., CUIJPERS, P., FUNK, B. & LEHR, D. 2016a. Self-guided internet-based and mobile-based stress management for employees: results of a randomised controlled trial. *Occup Environ Med,* 73**,** 315-23.

EBERT, D. D., LEHR, D., BOSS, L., RIPER, H., CUIJPERS, P., ANDERSSON, G., THIART, H., HEBER, E. & BERKING, M. 2014. Efficacy of an internet-based problem-solving training for teachers: results of a randomized controlled trial. *Scand J Work Environ Health,* 40**,** 582-96.

EBERT, D. D., LEHR, D., HEBER, E., RIPER, H., CUIJPERS, P. & BERKING, M. 2016b. Internet- and mobile-based stress management for employees with adherence-focused guidance: efficacy and mechanism of change. *Scand J Work Environ Health,* 42**,** 382-94.

ERIKSSON, T., GERMUNDSJO, L., ASTROM, E. & RONNLUND, M. 2018. Mindful Self-Compassion Training Reduces Stress and Burnout Symptoms Among Practicing Psychologists: A Randomized Controlled Trial of a Brief Web-Based Intervention. *Front Psychol,* 9**,** 2340.

FEICHT, T., WITTMANN, M., JOSE, G., MOCK, A., VON HIRSCHHAUSEN, E. & ESCH, T. 2013. Evaluation of a seven-week web-based happiness training to improve psychological well-being, reduce stress, and enhance mindfulness and flourishing: a randomized controlled occupational health study. *Evid Based Complement Alternat Med,* 2013**,** 676953.

GERAEDTS, A. S., KLEIBOER, A. M., TWISK, J., WIEZER, N. M., VAN MECHELEN, W. & CUIJPERS, P. 2014a. Long-term results of a web-based guided self-help intervention for employees with depressive symptoms: randomized controlled trial. *J Med Internet Res,* 16**,** e168.

GERAEDTS, A. S., KLEIBOER, A. M., WIEZER, N. M., VAN MECHELEN, W. & CUIJPERS, P. 2014b. Short-term effects of a web-based guided self-help intervention for employees with depressive symptoms: randomized controlled trial. *J Med Internet Res,* 16**,** e121.

GLUCK, T. M. & MAERCKER, A. 2011. A randomized controlled pilot study of a brief web-based mindfulness training. *BMC Psychiatry,* 11**,** 175.

GOLLWITZER, P. M., MAYER, D., FRICK, C. & OETTINGEN, G. 2018. Promoting the Self-Regulation of Stress in Health Care Providers: An Internet-Based Intervention. *Front Psychol,* 9**,** 838.

GRIME, P. R. 2004. Computerized cognitive behavioural therapy at work: a randomized controlled trial in employees with recent stress-related absenteeism. *Occup Med (Lond),* 54**,** 353-9.

GUILLE, C., ZHAO, Z., KRYSTAL, J., NICHOLS, B., BRADY, K. & SEN, S. 2015. Web-Based Cognitive Behavioral Therapy Intervention for the Prevention of Suicidal Ideation in Medical Interns: A Randomized Clinical Trial. *JAMA Psychiatry,* 72**,** 1192-8.

HAMAMURA, T., SUGANUMA, S., UEDA, M., MEARNS, J. & SHIMOYAMA, H. 2018. Standalone Effects of a Cognitive Behavioral Intervention Using a Mobile Phone App on Psychological Distress and Alcohol Consumption Among Japanese Workers: Pilot Nonrandomized Controlled Trial. *JMIR Ment Health,* 5**,** e24.

HASSON, D., ANDERBERG, U. M., THEORELL, T. & ARNETZ, B. B. 2005. Psychophysiological effects of a web-based stress management system: a prospective, randomized controlled intervention study of IT and media workers [ISRCTN54254861]. *BMC Public Health,* 5**,** 78.

HEBER, E., LEHR, D., EBERT, D. D., BERKING, M. & RIPER, H. 2016. Web-Based and Mobile Stress Management Intervention for Employees: A Randomized Controlled Trial. *J Med Internet Res,* 18**,** e21.

HERSCH, R. K., COOK, R. F., DEITZ, D. K., KAPLAN, S., HUGHES, D., FRIESEN, M. A. & VEZINA, M. 2016. Reducing nurses' stress: A randomized controlled trial of a web-based stress management program for nurses. *Appl Nurs Res,* 32**,** 18-25.

IMAMURA, K., FURUKAWA, T. A., MATSUYAMA, Y., SHIMAZU, A., KURIBAYASHI, K., KASAI, K. & KAWAKAMI, N. 2018. Differences in the Effect of Internet-Based Cognitive Behavioral Therapy for Improving Nonclinical Depressive Symptoms Among Workers by Time Preference: Randomized Controlled Trial. *J Med Internet Res,* 20**,** e10231.

IMAMURA, K., KAWAKAMI, N., FURUKAWA, T. A., MATSUYAMA, Y., SHIMAZU, A., UMANODAN, R., KAWAKAMI, S. & KASAI, K. 2014. Effects of an Internet-based cognitive behavioral therapy (iCBT) program in Manga format on improving subthreshold depressive symptoms among healthy workers: a randomized controlled trial. *PLoS One,* 9**,** e97167.

IMAMURA, K., KAWAKAMI, N., FURUKAWA, T. A., MATSUYAMA, Y., SHIMAZU, A., UMANODAN, R., KAWAKAMI, S. & KASAI, K. 2015. Does Internet-based cognitive behavioral therapy (iCBT) prevent major depressive episode for workers? A 12-month follow-up of a randomized controlled trial. *Psychol Med,* 45**,** 1907-17.

IMAMURA, K., KAWAKAMI, N., TSUNO, K., TSUCHIYA, M., SHIMADA, K. & NAMBA, K. 2016. Effects of web-based stress and depression literacy intervention on improving symptoms and knowledge of depression among workers: A randomized controlled trial. *J Affect Disord,* 203**,** 30-37.

JONAS, B., LEUSCHNER, F. & TOSSMANN, P. 2017. Efficacy of an internet-based intervention for burnout: a randomized controlled trial in the German working population. *Anxiety Stress Coping,* 30**,** 133-144.

KETELAAR, S. M., NIEUWENHUIJSEN, K., GARTNER, F. R., BOLIER, L., SMEETS, O. & SLUITER, J. K. 2013. Effect of an E-mental health approach to workers' health surveillance versus control group on work functioning of hospital employees: a cluster-RCT. *PLoS One,* 8**,** e72546.

LAPPALAINEN, P., KAIPAINEN, K., LAPPALAINEN, R., HOFFREN, H., MYLLYMAKI, T., KINNUNEN, M. L., MATTILA, E., HAPPONEN, A. P., RUSKO, H. & KORHONEN, I. 2013. Feasibility of a personal health technology-based psychological intervention for men with stress and mood problems: randomized controlled pilot trial. *JMIR Res Protoc,* 2**,** e1.

LILLY, M., CALHOUN, R., PAINTER, I., BEATON, R., STANGENES, S., REVERE, D., BASEMAN, J. & MEISCHKE, H. 2019. Destress 9-1-1—an online mindfulness-based intervention in reducing stress among emergency medical dispatchers: a randomised controlled trial. *Occupational and Environmental Medicine,* 76**,** 705.

LY, K. H., ASPLUND, K. & ANDERSSON, G. 2014. Stress management for middle managers via an acceptance and commitment-based smartphone application: A randomized controlled trial. *Internet Interventions,* 1**,** 95-101.

MAK, W. W., CHAN, A. T., CHEUNG, E. Y., LIN, C. L. & NGAI, K. C. 2015. Enhancing Web-based mindfulness training for mental health promotion with the health action process approach: randomized controlled trial. *J Med Internet Res,* 17**,** e8.

MISTRETTA, E. G., DAVIS, M. C., TEMKIT, M., LORENZ, C., DARBY, B. & STONNINGTON, C. M. 2018. Resilience Training for Work-Related Stress Among Health Care Workers: Results of a Randomized Clinical Trial Comparing In-Person and Smartphone-Delivered Interventions. *J Occup Environ Med,* 60**,** 559-568.

MORI, M., TAJIMA, M., KIMURA, R., SASAKI, N., SOMEMURA, H., ITO, Y., OKANOYA, J., YAMAMOTO, M., NAKAMURA, S. & TANAKA, K. 2014. A web-based training program using cognitive behavioral therapy to alleviate psychological distress among employees: randomized controlled pilot trial. *JMIR Res Protoc,* 3**,** e70.

OISHI, S., TAKIZAWA, T., KAMATA, N., MIYAJI, S., TANAKA, K. & MIYAOKA, H. 2018. Web-Based Training Program Using Cognitive Behavioral Therapy to Enhance Cognitive Flexibility and Alleviate Psychological Distress Among Schoolteachers: Pilot Randomized Controlled Trial. *JMIR Res Protoc,* 7**,** e32.

PERSSON ASPLUND, R., DAGÖÖ, J., FJELLSTRÖM, I., NIEMI, L., HANSSON, K., ZERAATI, F., ZIUZINA, M., GERAEDTS, A., LJÓTSSON, B., CARLBRING, P. & ANDERSSON, G. 2018. Internet-based stress management for distressed managers: results from a randomised controlled trial. *Occup Environ Med,* 75**,** 105-113.

PHILLIPS, R., SCHNEIDER, J., MOLOSANKWE, I., LEESE, M., FOROUSHANI, P. S., GRIME, P., MCCRONE, P., MORRISS, R. & THORNICROFT, G. 2014. Randomized controlled trial of computerized cognitive behavioural therapy for depressive symptoms: effectiveness and costs of a workplace intervention. *Psychol Med,* 44**,** 741-52.

PRASEK, A. 2015. *Randomized Controlled Trial to Evaluate a Self-Guided, Web-Based Mindfulness Program for Stress Reduction and Wellbeing Promotion.* Doctor of Philosophy, University of Minnesota.

QUERSTRET, D., CROPLEY, M. & FIFE-SCHAW, C. 2018. The Effects of an Online Mindfulness Intervention on Perceived Stress, Depression and Anxiety in a Non-clinical Sample: A Randomised Waitlist Control Trial. *Mindfulness (N Y),* 9**,** 1825-1836.

RUWAARD, J., LANGE, A., BOUWMAN, M., BROEKSTEEG, J. & SCHRIEKEN, B. 2007. E-mailed standardized cognitive behavioural treatment of work-related stress: a randomized controlled trial. *Cogn Behav Ther,* 36**,** 179-92.

SHIMAZU, A., KAWAKAMI, N., IRIMAJIRI, H., SAKAMOTO, M. & AMANO, S. 2005. Effects of web-based psychoeducation on self-efficacy, problem solving behavior, stress responses and job satisfaction among workers: a controlled clinical trial. *J Occup Health,* 47**,** 405-13.

SHIROTSUKI, K., NONAKA, Y., ABE, K., ADACHI, S. I., ADACHI, S., KUBOKI, T. & NAKAO, M. 2017. The effect for Japanese workers of a self-help computerized cognitive behaviour therapy program with a supplement soft drink. *Biopsychosoc Med,* 11**,** 23.

SONG, M. & KANAOKA, H. 2018. Effectiveness of mobile application for menstrual management of working women in Japan: randomized controlled trial and medical economic evaluation. *J Med Econ,* 21**,** 1131-1138.

STANSFELD, S. A., KERRY, S., CHANDOLA, T., RUSSELL, J., BERNEY, L., HOUNSOME, N., LANZ, D., COSTELLOE, C., SMUK, M. & BHUI, K. 2015. Pilot study of a cluster randomised trial of a guided e-learning health promotion intervention for managers based on management standards for the improvement of employee well-being and reduction of sickness absence: GEM Study. *BMJ Open,* 5**,** e007981.

STRATTON, E., CHOI, I., CALVO, R., HICKIE, I., HENDERSON, C., HARVEY, S. B. & GLOZIER, N. 2019. Web-based decision aid tool for disclosure of a mental health condition in the workplace: a randomised controlled trial. *Occup Environ Med,* 76**,** 595-602.

SUZUKI, E., TSUCHIYA, M., HIROKAWA, K., TANIGUCHI, T., MITSUHASHI, T. & KAWAKAMI, N. 2008. Evaluation of an internet-based self-help program for better quality of sleep among Japanese workers: a randomized controlled trial. *J Occup Health,* 50**,** 387-99.

UMANODAN, R., SHIMAZU, A., MINAMI, M. & KAWAKAMI, N. 2014. Effects of computer-based stress management training on psychological well-being and work performance in japanese employees: a cluster randomized controlled trial. *Ind Health,* 52**,** 480-91.

VILLANI, D., GRASSI, A., COGNETTA, C., TONIOLO, D., CIPRESSO, P. & RIVA, G. 2013. Self-help stress management training through mobile phones: an experience with oncology nurses. *Psychol Serv,* 10**,** 315-322.

VOLKER, D., ZIJLSTRA-VLASVELD, M. C., ANEMA, J. R., BEEKMAN, A. T., BROUWERS, E. P., EMONS, W. H., VAN LOMWEL, A. G. & VAN DER FELTZ-CORNELIS, C. M. 2015. Effectiveness of a blended web-based intervention on return to work for sick-listed employees with common mental disorders: results of a cluster randomized controlled trial. *J Med Internet Res,* 17**,** e116.

WEBER, S., LORENZ, C. & HEMMINGS, N. 2019. Improving Stress and Positive Mental Health at Work via an App-Based Intervention: A Large-Scale Multi-Center Randomized Control Trial. *Front Psychol,* 10**,** 2745.

YAMAGISHI, M., KOBAYASHI, T. & NAKAMURA, Y. 2008. Effects of web-based career identity training for stress management among Japanese nurses: a randomized control trial. *J Occup Health,* 50**,** 191-3.

YUAN, Q. 2015. *Evaluating the Effectiveness of A Psychological Capital Development Program on Mental Health, Engagement and Work Performance.* Doctor of Philosophy PhD, The Chinese University of Hong Kong.

ZHANG, H., JIANG, Y., NGUYEN, H. D., POO, D. C. & WANG, W. 2017. The effect of a smartphone-based coronary heart disease prevention (SBCHDP) programme on awareness and knowledge of CHD, stress, and cardiac-related lifestyle behaviours among the working population in Singapore: a pilot randomised controlled trial. *Health Qual Life Outcomes,* 15**,** 49.

ZWERENZ, R., BECKER, J., GERZYMISCH, K., SIEPMANN, M., HOLME, M., KIWUS, U., SPORL-DONCH, S. & BEUTEL, M. E. 2017. Evaluation of a transdiagnostic psychodynamic online intervention to support return to work: A randomized controlled trial. *PLoS One,* 12**,** e0176513.
