# Supplementary figures and images for: Trends in Effectiveness of Organizational eHealth Interventions in Addressing Employee Mental Health: Systematic Review and Meta-analysis
Source: J Med Internet Res. 2022 Sep 27;24(9):e37776. doi: 10.2196/37776 (PMC9555335; doi:10.2196/37776)

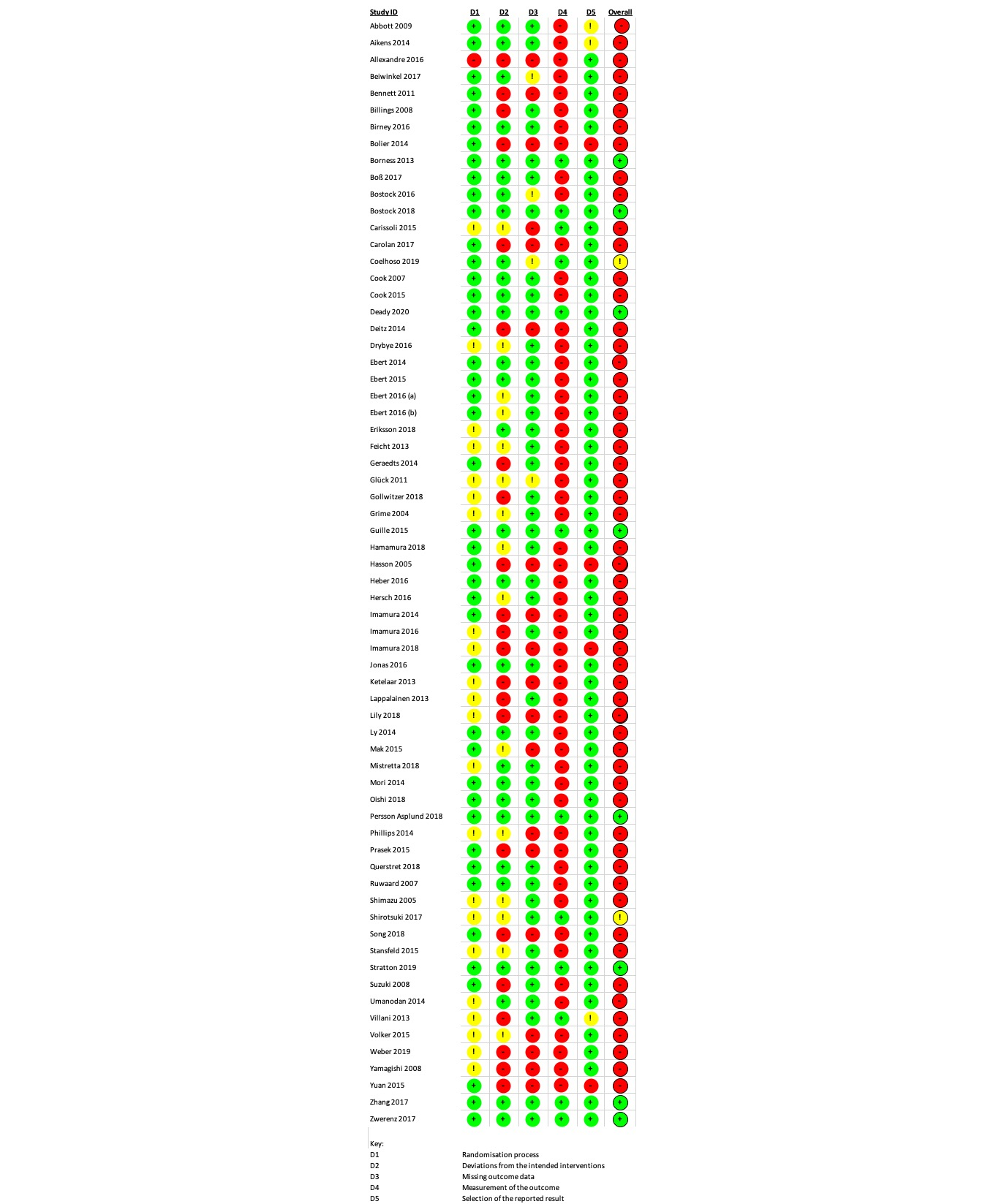

Supplement: Multimedia Appendix 3 [file jmir_v24i9e37776_app3.png]

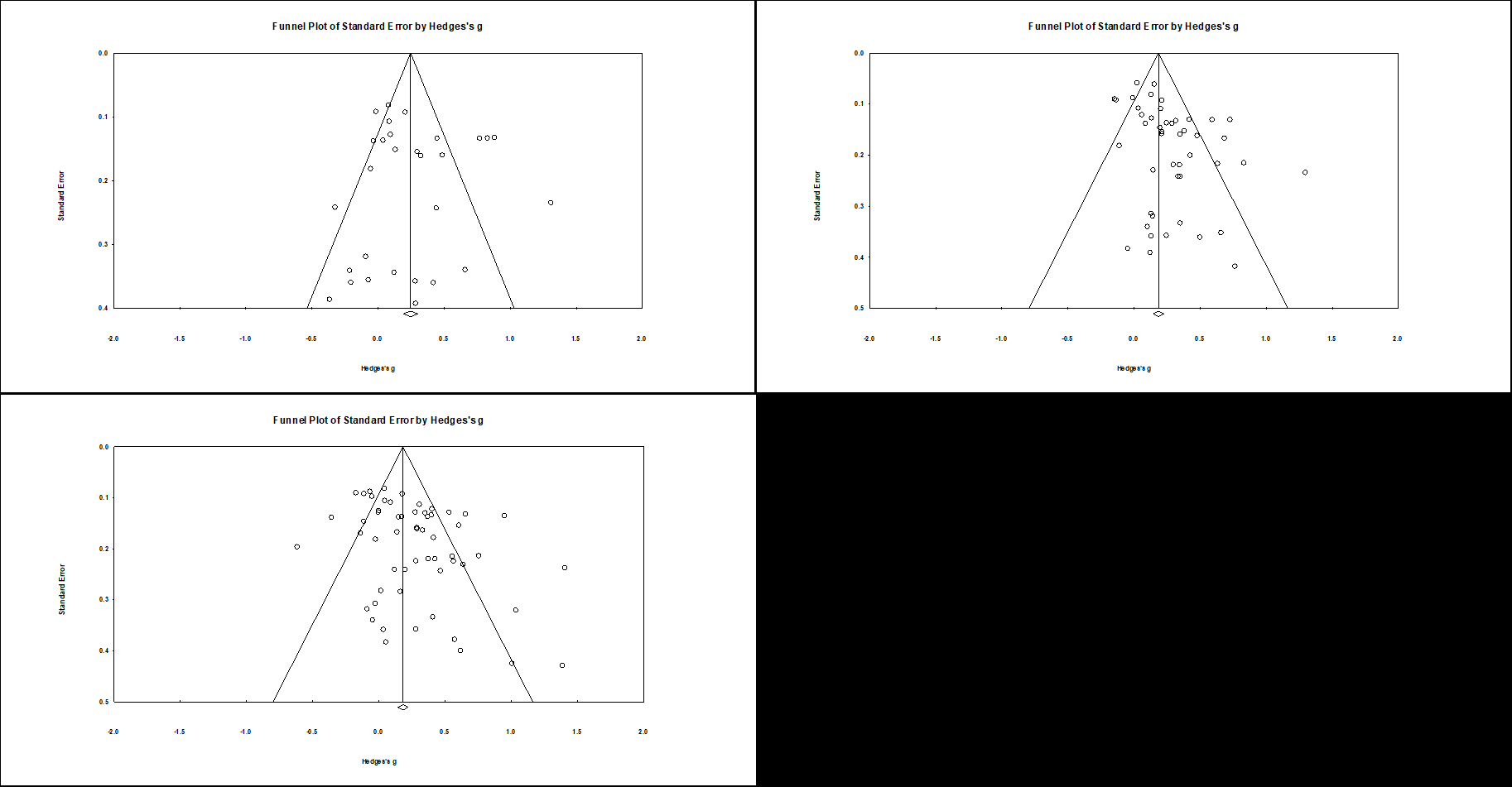

Supplement: Multimedia Appendix 6 [file jmir_v24i9e37776_app6.png]
