# Supplementary material for: Trends in Effectiveness of Organizational eHealth Interventions in Addressing Employee Mental Health: Systematic Review and Meta-analysis
Source: J Med Internet Res. 2022 Sep 27;24(9):e37776. doi: 10.2196/37776 (PMC9555335; doi:10.2196/37776)
Supplement: Multimedia Appendix 4 [file jmir_v24i9e37776_app4.docx]

**Multimedia Appendix 4 Mental health measures**

| Anxiety Measure | Times Used | Depression Measure | Times Used | Stress Measure | Times Used |
| --- | --- | --- | --- | --- | --- |
| DASS21-42 | 8 | PHQ-2-9 | 10 | PSS-10-14 | 15 |
| HADS-A | 6 | CES-D | 9 | K6 | 6 |
| GAD-7 | 5 | DASS-21-42 | 7 | DASS-21-42 | 6 |
| STAI | 2 | BDI | 5 | PSQ | 3 |
| STPI | 1 | MADRS | 1 | SDS | 2 |
| GAD-2 | 1 | BSI | 1 | BJSQ | 2 |
| POMS-A | 1 | JSBQ | 1 | VAS | 1 |
| BAI | 1 | PRIME MD | 1 | JSBQ | 1 |
| BSI | 1 | POMS-D | 1 | SWS | 1 |
| JSBQ | 1 | STPI | 1 | CWS | 1 |
|  |  |  |  | MSP | 1 |
|  |  |  |  | GHQ-12 | 1 |
|  |  |  |  | NSS | 1 |
|  |  |  |  | C-SOSI | 1 |
|  |  |  |  | COPSOQ II | 1 |
|  |  |  |  | DSQ | 1 |
|  |  |  |  | BBI | 1 |
