# Supplementary material for: Trends in Effectiveness of Organizational eHealth Interventions in Addressing Employee Mental Health: Systematic Review and Meta-analysis
Source: J Med Internet Res. 2022 Sep 27;24(9):e37776. doi: 10.2196/37776 (PMC9555335; doi:10.2196/37776)
Supplement: Multimedia Appendix 5 [file jmir_v24i9e37776_app5.docx]

**Multimedia Appendix 5. Detailed description of studies selected**

| **Study (name, year)** | **Type** | **Targeted to mental health symptoms or not** | **Participants** | **Intervention, Duration + n** | **Control, n** | **Type of intervention** | **Primary Outcome specified in study** | **Mental health Outcome(s) + Measure(s)** | **Baseline score (m, SD, n)** | **Post- intervention from baseline** | **Results – Post- intervention (m, SD, n)** | **Follow-up from baseline** | **Results - Follow- up** |
| --- | --- | --- | --- | --- | --- | --- | --- | --- | --- | --- | --- | --- | --- |
| Grime (2004) * | CBT | Yes, depression | UK, Occupational Health department. Employees with >10 days sick leave in the past 6 months due to mental illness (n=48) | 'Beating the Blues' a computerized CBT program for depression and anxiety, 8 online sessions which last approximately an hour per week (n=24) | WLC (n=24) | Tertiary | Depression | Anxiety & Depression (HADS) | Intervention:  Depression (7.96,3.43,24) Anxiety (11.75,3.87,24)   Control:  Depression (10.63,4.13,24) Anxiety (14.04,4.34,24) | 2 months | Depression  Intervention (5.38,3.93,16)  Control (8.61,3.86,23)  Anxiety  Intervention (10.13,4.65,14) Control (12.00,4.31,23) | 3 months | Depression  Intervention (5.00,4.12,13)  Control (7.32,5.08,19)  Anxiety  Intervention (8.69,3.50,13) Control (9.47,5.26,19) |
|  |  |  |  |  |  |  |  |  |  |  |  | 6 months | Depression  Intervention (5.07,4.57,14)  Control (6.21,4.22,19)  Anxiety  Intervention (8.86,4.35,14)  Control (9.16,4.37,19) |
| Hasson (2005) * ^ | CBT | No | Sweden, IT, and media companies’ general employees (n=317) | 12-month open access. Web-based self-help exercises - developed specifically for this study. Uses CBT techniques (n=129) | WLC + info (n=174) | Universal | Stress | Stress (VAS) | Intervention:  Stress (129)   Control:  Stress (174) | 12 months | Stress  Intervention (121) Control (156) | nil | not measured |
| Shimazu (2005) * | CBT | No | Japan, any employee company wide in a construction machinery company (n=225) | One-month web-based psychoeducation based on social cognitive theory. Self- based program, 3 phases (5 chapters), (n=112) | WLC (n=113) | Universal | Self-efficacy & Problem-Solving Behavior | Stress (BJSQ) | Intervention:  Stress (36.80,9.88,112)   Control:  Stress (38.30,9.39,113) | 5 weeks | Stress  Intervention (35.70,5.68,105) Control (37.60,6.76,107) | nil | not measured |
| Cook (2007) * | Stress Mx | No | USA, human resource employees (n=419) | Web-based multimedia health promotion program 'Health Connection' for the workplace offering guidance on stress management (n=209) | WLC + paper-based info (n=210) | Universal | Dietary Practices | Stress (PSS) | Intervention:  Stress (14.21,4.90,209)  Control: Stress (15.05,4.60,210) | 3 months | Stress  Intervention (13.70,4.90,209) Control (14.30,4.50,210) | nil | not measured |
| Ruwaard (2007) * ^ | CBT | Both general and symptomatic | Netherlands, general employees (n=239) | 7-week online CBT. Supported by trained therapists with 10 personalized feedback sessions. One module per week, awareness, relaxation, worrying, positive self-verbalization, positive assertiveness, time management. In person and audio training. (n=177) | WLC (n=62) | Tailored | Stress | Stress (DASS-42) | Intervention:  Depression (11.80,6.70,177) Anxiety (8.20,6.20,177) Stress (19.40,8.00,177)  Control: Depression (12.10,7.30,62) Anxiety (9.60,6.70,62) Stress (19.70,8.00,62) | 7 weeks | Depression  Intervention (7.80,6.60,150) Control (10.70,7.30,61)  Anxiety Intervention (5.20,5.20,150) Control (7.30,5.50,61)  Stress Intervention (12.00,8.50,150) Control (17.60,9.30,61) | nil | not measured |
| Billings (2008) # | CBT | Both general and symptomatic | USA, employees from a technology company on a health and activity promotion program (n=309) | 3-month web-based. Participants could review sections more than once. Uses CBT - website is tailored around baseline answers for each participant depending on risks and needs (n=154) | WLC (n=155) | Tailored | Psychological -Knowledge, Attitude and Self-efficacy | Stress (SDS), Depression (CES-D),  Anxiety (BAI) | Intervention:  Stress (17.52,4.53,154) Depression (33.77,13.46,115) Anxiety (28.87,7.82,154)  Control: Stress (16.81,3.78,155) Depression (32.15,10.08,155) Anxiety (27.98,7.43,155) | 3 months | Stress  Intervention (16.03,4.18,113) Control (16.50,4.35,132)  Depression  Intervention (31.60,13.33,113) Control (31.57,10.56,132)  Anxiety  Intervention (27.54,7.53,113) Control (27.30,6.19,132) | nil | not measured |
| Suzuki (2008) * | CBT | No | Japan, general university staff (n=43) | 2-week program, four modules about improving sleep quality. Daily monitoring and feedback and sleep diary. Emailed weekly summary and advice (n=21) | WLC (n=22) | Universal | Sleep quality | Stress (K6) | Intervention:  Stress (5.08,3.42,21)  Control: Stress (7.29,4.55,22) | 1 month | Stress  Intervention (5.08,3.99,19) Control (7.17,4.71,22) | nil | not measured |
| Yamagishi (2008) * | Assertion training | No | Japanese shift working Nurses (n=60) | 9 weeks sixty-minute web-based training was provided weekly (n=30) | WLC (n=30) | Universal | Stress | Stress, Anxiety, Depression (JSBQ) | Intervention:  Stress (2.54,0.67,30) Depression (1.85,0.70,30) Anxiety (2.18,0.80,30)  Control:  Stress (2.35,0.80,30) Depression (1.89,0.90,30) Anxiety (2.03,0.80,30) | 5 weeks | Stress  Intervention (2.58,0.60,30) Control (2.79,0.80,30)  Depression  Intervention (1.93,0.70,30) Control (2.04,0.80,30)  Anxiety  Intervention (2.26,0.70,30) Control (2.33,0.80,30) | 9 weeks | Stress  Intervention (2.60,0.70,30) Control (2.67,0.70,30)  Depression  Intervention (1.71,0.50,30) Control (1.78,0.60,30)  Anxiety  Intervention (2.00,0.69,30) Control (2.10,0.80,30) |
| Abbott (2009) * | CBT | No | Australia, Industrial organization general employees (n=53) | Internet-based program teaching resilience through cognitive therapy. 7 core modules (n=26) | WLC (n=27) | Universal | Happiness | Depression, Anxiety, Stress (DASS-21) | Intervention:  Stress (10.70,7.40,26) Depression (5.30,5.10,26) Anxiety (2.30,2.20,26)  Control:  Stress (7.60,6.90,27) Depression (3.78,5.40,27) Anxiety (2.07,2.80,27) | 10 weeks | Stress  Intervention (9.70,6.00,26) Control (1.56,2.40,27)  Depression  Intervention (4.70,4.90,26) Control (3.70,5.30,27)  Anxiety  Intervention (2.30,2.20,26) Control (6.30,4.90,27) | nil | not measured |
| Bennett (2011) * | Behavior Change | No | USA, general Managers (n=145) | Internet-based program tested in the study, ExecuPrev™, trained managers to modify attitudes and behaviors’, and built motivation to be healthy and effective leaders. Users were instructed to spend at least 10 hours during the 6-month access (n=72) | WLC (n=73) | Universal | Cardio-vascular health | Stress (SDS) | Intervention:  Stress (3.92,0.18,72)  Control: Stress (3.70,0.17,73) | 6 months | Stress  Intervention (4.28,0.21,47) Control (3.94,0.18,62) | nil | not measured |
| Glück (2011) * | Mindfulness | No | Austria, Germany & Switzerland General employees of universities, car dealership, broadcasting station, and health care consulting companies in (n=50) | web based mindfulness training 13 days two modules each module lasted for 6 days with 20 mins per day (n=28) | WLC (n=21) | Universal | Distress | Distress (BSI), Stress (PSQ) | Intervention:  Stress (40.06,16.40,28)  Control: Stress (35.10,13.40,21) | 2 weeks | Stress  Intervention (34.40,15.00,26) Control (34.72,15.35,18) | 3 months | Stress  Intervention (27.90,11.20,19) Control group not tested |
| Borness (2013) * | Cognitive training | No | Australia, Public Sector general employees (n=135) | 16 weeks of online Cognitive Training based around, memory, attention, language, executive function, program is called 'Spark!' with three 20-minute sessions per week (n=67) | active control program (n=68) general knowledge information | Universal | Stress & Quality of life | Stress (JSS), Depression, Anxiety, (DASS- 42) | Intervention:  Stress (10.70,6.70,67) Depression (6.20,6.20,67) Anxiety (5.40,5.60,67)  Control: Stress (11.00,8.80,68) Depression (7.40,6.80,68) Anxiety (6.60,5.80,68) | 4 months | Stress  Intervention (10.90,7.90,58) Control (5.80,8.20,62)  Depression  Intervention (7.30,6.90,58) Control (6.80,7.60,62)  Anxiety  Intervention (6.20,7.10,58) Control (6.60,11.50,62) | nil | not measured |
| Feicht (2013) * | Positive Psychology | No | Germany, general employees in an insurance company (n=147) | 7-week online happiness training. Weekly modules that took 10-15 mins. Email instructions once weekly to describe how to do the weekly module (n=85) | WLC (n=62) | Universal | Well-being & Stress | Stress (SWS) | Intervention:  Stress (184.80,92.27,85)  Control:  Stress (207.60,112.91,62) | 2 months | Stress  Intervention (147.28,87.57,72) Control (212.32,117.32,57) | 3 months | Stress  Intervention (133.09,74.64,68) Control (217.74,125.26,51) |
| Ketelaar (2013) * | Health Surveillance Model | Both general and symptomatic | Netherlands, nurses, and allied health professional (n=367) | The trajectories offered for improvement of mental health will be tailored to the needs of the worker as assessed by the screening. The e-mental health interventions offered are: Psyfit: aimed at the promotion of wellbeing. It is suitable for everyone, including healthy participants.  Strong at work: aimed at gaining insight into work stress and at learning skills to cope with work stress. Color your life: aimed at tackling depressive symptoms.   Don't Panic Online: aimed at reducing panic symptoms for sub-clinical and mild cases of panic disorder.  Drinking less: aimed at reducing risky alcohol drinking behavior (n=178) | WLC (n=188) | Tailored | Work function | Distress (4DSQ), PTSD (Impact of Event Scale) | Intervention:  Stress (6.88,6.52,178)  Control: Stress (7.14,7.20,188) | 3 months | Stress  Intervention (4.50,5.72,82) Control (5.66,6.18,144) | 6 months | Stress  Intervention (5.14,5.97,71) Control (5.91,6.39,138) |
| Lappalainen (2013) * ^ | CBT & ACT | Yes, depression | Finland, males aged 28-58yrs with depression (n=23) | ‘P4Well', 3-month novel CBT intervention Delivered via multiple channels, including 3 group meetings, Internet/Web portal, mobile phone applications, and personal monitoring devices. (n=11) | WLC (n=12) | Tertiary | Depression, Stress, Psychological symptoms | Depression (BDI 21-item), Psychological symptom (GSI), Stress (BBI-15) | Intervention:  Depression (14.64,7.61,11)  Control:  Depression (13.33,9.24,12) | 3 months | Depression  Intervention (6.20,3.30,11) Control (9.30,7.10,12) | 6 months | Depression  Intervention (6.20,3.30,11) Control not measured |
| Villani (2013) *^ | Stress Mx | Yes, High stress | Italy, Female oncology nurses with high stress (n=30) | 4-week intervention protocol included eight 5-minute video clips twice weekly with a narrative. After work on study phone (n=15) | Active control eight video clips at the same time as the intervention arm of neutral stimuli (n=15) | Indicated | Anxiety | Anxiety (STAI) | Intervention:  Anxiety (43.64,8.03,15)  Control: Anxiety (44.00,9.91,15) | 1 month | Anxiety  Intervention (41.00,8.80,15) Control (42.14,11.67,15) | nil | not measured |
| Bolier (2014) * ^ | Health Surveillance Model | Both general and symptomatic | Netherland, Nurses, and allied health professionals’ general employees (n=423) | Tailored to the needs of the worker as assessed by the screening. Interventions offered are: Psyfit: aimed at the promotion of wellbeing. It is suitable for everyone, including healthy participants.  Strong at work: aimed at gaining insight into work stress and at learning skills to cope with work stress. Color your life: aimed at tackling depressive symptoms.   Don't Panic Online: aimed at reducing panic symptoms for sub-clinical and mild cases of panic disorder.  Drinking less: aimed at reducing risky alcohol drinking behavior  Based on screening results participants were offered a tailored web-based intervention ranging from 4 to 8 weeks (n=212) | WLC (n=211) | Tailored | Positive Mental Health | Depression (BSI), Anxiety (BSI) | Intervention: Depression (0.31,0.32,212) Anxiety (0.21,0.24,212)  Control:  Depression (0.30,0.35,212) Anxiety (0.25,0.32,211) | 3 months | Depression  Intervention (0.24,0.32,143) Control (0.26,0.35,82)  Anxiety  Intervention (0.16,0.24,143) Control (0.19,0.30,82) | 6 months | Depression Intervention (0.22,0.31,138) Control (0.29,0.35,70)  Anxiety  Intervention (0.17,0.24,138) Control (0.22,0.32,70) |
| Deitz (2014) * | Behavior Change | No | USA, hospital employees with cardiovascular risk (n=210) | 6-week web-based intervention for promoting cardiovascular health, weekly modules. Based around increasing knowledge and reducing risk (n=105) | WLC (n=105) | Universal | Cardio-vascular health | Stress (CWS), Depression and Anxiety (STPI) | Intervention: Depression (1.80,0.53,105) Stress (2.95,0.38,105)  Control: Depression (1.68,0.53,105) Stress (3.00,0.38,105) | 6 weeks | Depression  Intervention (1.70,0.53,105) Control (1.75,0.64,105)  Stress Intervention (3.06,0.40,105) Control (2.96,0.44,105) | nil | not measured |
| Ebert (2014) * ^ | Problem Solving Training | Yes, depression | Germany, teachers with elevated depressive symptoms currently employed (n=150) | 6 weeks, 5 lessons, based on problem solving techniques, internet-based problem-solving training (iPST), one lesson per week and practice problem solving skills between each lesson (n=75) | WLC (n=75) | Tertiary | Depression | Depression (CES-D), Stress (PSQ) | Intervention:  Stress (0.66,0.15,75) Depression (22.76,9.20,75)  Control:  Stress (0.67,0.14,75) Depression (22.80,9.15,75) | 7 weeks | Stress  Intervention (0.55,0.20,75) Control (0.62,0.17,75)  Depression  Intervention (22.81,9.15,75) Control (21.20,8.37,75) | 3 months | Stress  Intervention (0.48,0.27,75) Control (0.56,0.25,75)  Depression  Intervention (15.37,8.40,75) Control (19.87,14.00,75) |
|  |  |  |  |  |  |  |  |  |  |  |  | 6 months | Stress  Intervention (0.53,0.20,75) Control (0.60,0.16,75)  Depression  Intervention (14.53,13.80,75) Control (19.91,10.42,75) |
| Geraedts (2014) * ^ (two papers) | Problem Solving Training & Cognitive Therapy | Yes, depression | Netherland, employees from banking companies with elevated depression (n=231) | Web-based guided self-help intervention, called Happy@Work, 6 weekly sessions consisted of problem-solving techniques and CBT, complete an assessment each week to move on (n=116) | WLC (n=115) | Tertiary | Depression | Depression (CES-D), Anxiety (HADS) | Intervention:  Depression (25.70,7.50,116) Anxiety (10.60,3.80,116)  Control:  Depression (26.10,7.00,116) Anxiety (10.20,3.20,116) | 2 months | Depression  Intervention (15.80,10.60,116) Control (18.30,9.10,115)  Anxiety  Intervention (7.60,3.80,116) Control (8.30,3.60,115) | 12 months | Depression  Intervention (15.70,11.30,116) Control (16.20,10.70,115)  Anxiety  Intervention (6.80,4.10,116) Control (6.80,4.00,115) |
| Imamura (2014) & (2015) * ^ (two papers) | CBT | Yes, depression | Japan, IT employees with Major Depressive Disorder in last month or treated for mental health problems in last month (n=762) | 6-week internet-based computerized CBT. 6 lessons, one lesson per week, approx. 30 mins each. Each lesson had homework. Given 10 weeks to complete study in whole (n=381) | WLC + info (n=381) | Tertiary | Depression | Depression (BDI-21), Stress (K6), anxiety (DASS) | Intervention:  Stress (5.60,4.60,381) Depression (11.90,8.00,381) Anxiety (88.00,21.50,381)  Control: Stress (5.60,4.70,381) Depression (11.80,8.00,381) Anxiety (87.40,20.90,381) | 3 months | Stress  Intervention (5.60,4.60,270) Control (5.80,4.70,336)  Depression  Intervention (10.70,8.60,270) Control (11.70,8.30,336)  Anxiety  Intervention (87.40,21.70,270) Control (88.50,20.90,336) | 6 months | Stress  Intervention (5.70,4.80,272) Control (6.40,5.10,320)  Depression  Intervention (11.30,9.60,272) Control (12.10,8.70,320)  Anxiety  Intervention (84.90,23.30,272) Control (87.00,22.80,320) |
|  |  |  |  |  |  |  |  |  |  |  |  | 12 months | Stress  Intervention (5.40,4.70,239) Control (5.80,4.70,272)  Depression  Intervention (11.20,9.40,239) Control (11.60,9.00,272) |
| Ly (2014) *^ | ACT | No | Sweden, middle managers or have staff responsibilities in the private sector (n=73) | 6-week smartphone app mindfulness based. 6 modules one for each week. each module had an audio lecture, text & exercises, supported by student psychologist (n=36) | WLC (n=37) | Universal | Stress and Psychological Distress | Stress (PSS-14), Psychological Distress (GHQ-12) | Intervention:  Stress (24.30,8.30,36)  Control Stress (24.50,5.90,37) | 6 weeks | Stress  Intervention (19.50,7.30,36) Control (23.30,8.00,37) | nil | not measured |
| Mori (2014) * ^ | CBT | No | Japan, IT employees, system engineers with high computer literacy (n=168) | 4-week intervention with homework. A Web-Based Training Program using CBT. 150-minute group class - participants use web-based entries to log daily stresses (n=85) | WLC (n=83) | Universal | Psychological Distress | Psychological distress (K6) | Intervention:  Stress (4.70,4.50,85)   Control:  Stress (4.80,4.50,83) | 1 month | Stress  Intervention (4.20,4.90,85) Control (5.00,4.80,83) | 6 months | Stress  Intervention (4.60,5.10,85) Control (5.63,5.00,83) |
| Phillips (2014) * | CBT | Yes, depression | UK, transport, health, and communications sectors who reported mild - moderate depression and difficulty in some tasks at work (n=637) | 5-week web-based intervention 'MoodGYM' five one-hour long modules containing CBT skills for preventing and coping with depression (n=318) | WLC + info (n=319) | Tertiary | Work related performance | Depression (PHQ-9), Anxiety (GAD-7), Distress (CORE-10) | Intervention:  Stress (19.90,8.00,318) Depression (14.50,5.40,318) Anxiety (13,50.4.00,318)  Control: Stress (20,70.7.00,319) Depression (14.60,5.60,319) Anxiety (13.20,5.00,319) | 6 weeks | Stress  Intervention (16.00,9.10,171) Control (16.50,8.60,188)  Depression  Intervention (9.90,6.10,164) Control (10.20,6.00,176)  Anxiety  Intervention (9.50,6.00,166) Control (10.20,5.70,181) | 3 months | Stress  Intervention (15.00,10.10,102) Control (15.90,8.60,129)  Depression  Intervention (9.30,6.90,97) Control (10.30,6.90,122)  Anxiety  Intervention (8.40,6.40,98) Control (10.10,6.50,123) |
| Umanodan (2014) * | Stress Mx | No | Japan, general employees in a manufacturing company (n=266) | 6-week Computer based stress management training (SMT) 6 lessons (one per week) - self paced. 2-phased learning process (n=142) | WLC (n=121) | Universal | Psychological Distress, work engagement, satisfaction, and performance | Psychological distress (BJSQ) | Intervention:  Stress (2.00,0.50,142) Control: Stress (2.10,0.56,121) | 9 weeks | Stress  Intervention (1.90,0.45,142) Control (2.00,0.55,121) | 19 weeks | Stress  Intervention (2.00,0.04,142) Control (2.00,0.55,121) |
| Carissoli (2015) # | Mindfulness | No | Italy, general employees (n=56) | 3-week smartphone application Meditation participants had to practice two mindfulness meditations per day, lasting 15 minutes each, by listening to the guided or free (starting from the second week) meditation supported by the smartphone application “It's time to relax!” (n=20) | Music participants had to use their mobile device to listen to two pieces of relaxing music (chosen from a proposed list) per day, lasting about 15 minutes each, while doing nothing else (n=18) | Universal | Stress | Stress (MSP) | Intervention:  Stress (10.75,3.19,20)  Control: Stress (10.28,2.16,18) | 6 weeks | Stress  Intervention (10.60,3.89,20) Control (9.83,2.60,18) | nil | not measured |
| Cook (2015) * | Stress Mx | No | USA, Employees aged 50-68 years global IT company (n=278) | HealthyPast50' is a web-based. Open access for 3 months, 5 modules on stress and mood management, healthy eating, active lifestyle, and smoking (n=138) | WLC (n=140) | Universal | Stress, Diet and Exercise | Stress (Ultimate Stress Management, Self- Assessment, and Coping) | Intervention:  Stress (3.24,0.50,138)  Control: Stress (3.20,0.40,140) | 3 months | Stress  Intervention (3.30,0.50,109) Control (3.34,0.50,130) | nil | not measured |
| Ebert (2015) * | Behavior change | No | Germany, Techers with elevated symptoms of insomnia and work-related strain (n=128) | 6-week internet-based weekly sessions that aimed to promote healthy restorative behavior (n=64) | WLC (n=64) | Universal | Insomnia | Depression (CES-D) | Intervention: Depression (21.13,7.61,64)  Control: Depression (22.65,7.08,64) | 2 months | Depression  Intervention (13.17,6.85,49) Control (19.22,13.17,51) | nil | not measured |
| Guille (2015) * | CBT | No | USA, Medical Interns (n=199) | MoodGYM, was developed by staff at the National Institute for Mental Health Research at the Australian National University. The program consisted of 4 weekly web-based sessions lasting approximately 30 minutes each (n=100) | control participants received 4 weekly emails. All emails included information about the prevalence of depression and suicide among physicians (n=99) | Universal | Suicidal Ideation | Depression (PHQ-9), Suicidal ideation (PHQ-9) | Intervention:  Depression (2.78,4.06,100)  Control: Depression (2.69,2.94,99) | 3 months | Depression Intervention (4.80,4.80,88) Control (5.66,4.85,99) | 6 months | Depression Intervention (4.89,4.13,69) Control (5.73,4.19,84) |
|  |  |  |  |  |  |  |  |  |  |  |  | 12 months | Depression Intervention (4.98,4.99,69) Control (5.51,4.46,84) |
| Mak (2015) * | MBSR | No | China, University staff, general employees (n=321) | two arm intervention: 8-week online mindfulness training, one lesson per week that took 23-30 (n=107) | WLC (n=107) | Universal | Mindfulness | Stress (PSS-10), Depression, Anxiety (DASS- 21) | Intervention:  Stress (1.70,0.60,107) Depression (6.10,6.30,107) Anxiety (6.70,5.20,107)  Control:  Stress (1.63,0.70,53) Depression (5.47,7.60,53) Anxiety (6.20,8.00,53) | 2 months | Stress  Intervention (1.65,0.50,58) Control (1.62,0.70,48)  Depression  Intervention (5.70,6.80,58) Control (5.60,7.70,48)  Anxiety  Intervention (6.60,5.40,58) Control (5.70,7.30,48) | 3 months | Stress  Intervention (1.70,0.50,44) Control (1.63,0.70,24)  Depression  Intervention (6.80,7.20,44) Control (5.90,8.12,24)  Anxiety  Intervention (7.30,6.40,44) Control (5.70,7.60,24) |
|  |  |  |  | the second group had the identical training plus HAPA (health action process approach) to test enhanced efficacy (n=107) |  |  |  |  | Intervention:  Stress (1.80,0.60,107) Depression (6.95,7.70,107) Anxiety (7.50,6.60,107)  Control:  Stress (1.63,0.70,54) Depression (5.47,7.60,54) Anxiety (6.20,8.00,54) |  | Stress  Intervention (1.70,0.60,58) Control (1.62,0.70,48)  Depression  Intervention (6.50,7.40,58) Control (5.60,7.70,48)  Anxiety  Intervention (6.30,6.60,58) Control (5.70,7.30,48) |  | Stress  Intervention (1.70,0.60,37) Control (1.63,0.70,24)  Depression  Intervention (6.90,7.70,37) Control (5.90,8.12,24)  Anxiety  Intervention (6.50,6.60,37) Control (5.70,7.60,24) |
| Prasek (2015) * | Mindfulness | No | USA, general University employees (n=192) | 7 week self-guided, web-based mindfulness program Sherman Project (n=101) | WLC (n=91) | Universal | Stress | Stress (PSS-10) | Intervention:  Stress (17.00,7.00,91)  Control: Stress (17.00,6.51,101) | 2 months | Stress  Intervention (17.00,7.08,59) Control (16.00,7.39,84) | nil | not measured |
| Stansfeld (2015) *^ | Stress Mx | No | UK, manager, and general employees of the NHS Mental Health Trust (n=275) | Online team-based health promotion program based around understanding stress through a series of linked topics with case examples. Six fortnightly modules for 3 months (n=216) | WLC (n=59) | Universal | Employee wellbeing and sickness absence | Psychological distress (GHQ- 12) | Intervention:  Stress (2.80,3.50,216)  Control:  Stress (3.20,3.40,59) | 3 months | Stress  Intervention (2.90,3.50,216) Control (2.90,3.40,59) | nil | not measured |
| Volker (2015) * | CBT and PST | Yes, depression and anxiety | Netherlands, sick-listed employees visiting an occupational physician (OP) (n=773) | Return @ Work, online, 5 modules. Up to 16 sessions, the content of Return@Work was tailor-made to the individual employee, depending on the symptoms and cognitions about RTW of the employee, the total number of sessions ranged from 6 to 17 (n=131) | WLC (n=89) | Tertiary | Return to work | Depression (PHQ-9), Anxiety (GAD-7) | Intervention:  Depression (131) Anxiety (131)  Control: Depression (89) Anxiety (89) | 3 months | Depression  Intervention (92) Control (92)  Anxiety Intervention (66) Control (66) | 6 months | Depression  Intervention (88) Control (88)  Anxiety Intervention (70) Control (70) |
|  |  |  |  |  |  |  |  |  |  | 9 months | Depression  Intervention (73) Control (73)  Anxiety Intervention (64) Control (64) | 12 months | Depression  Intervention (74) Control (74)  Anxiety Intervention (57) Control (57) |
| Yuan (2015) * | PST | No | Hong Kong | Happy @ Work online four training sessions, components: 1) hope, through goal setting 2) efficacy, an expressive writing training, 3) optimism, the ABCDE model of ‘learned optimism’, and 4) resilience skills were trained (n=162) | WLC (n=159) | Universal | Psychological Capital | Depression (CES-D) | Intervention:  Depression (16.54,7.95,162)  Control: Depression (17.52,8.71,159) | 1 month | Depression  Intervention (15.27,0.77,67) Control (17.86,0.85,98) | 2 months | Depression  Intervention (18.69,0.95,61) Control (17.65,0.83,94) |
|  |  |  |  |  |  |  |  |  |  |  |  | 12 months | Depression  Intervention (15.47,0.83,55) Control (17.02,0.79,87) |
| Allexandre (2016) * | Mindfulness | No | USA, General employees from a corporate call centre (n=91) | 8-week web-based educational program based on mindfulness meditation. 1 session per week, audio guided. Daily articles available and two-email reminder sent (n=54) | WLC (n=37) | Universal | Stress | Stress (PSS-10) | Intervention:  Stress (25.60,5.40,54)  Control: Stress (25.40,5.70,13) | 2 months | Stress  Intervention (19.80,7.60,30) Control (24.00,7.20,9) | 4 months | Stress  Intervention (19.40,7.70,27) Control (22.50,7.20,7) |
|  |  |  |  | access to above, plus met in groups of 11 to 12 people for 1 hour once a week for the 8-week duration of the online program. Group session included deep breathing exercise for 2mins, 10-mins audio recording, 20 to 30 min guided meditation and 20 min of discussion questions (n=37) |  |  |  |  | Intervention:  Stress (24.50,5.80,37)  Control: Stress (25.40,5.70,12) |  | Stress  Intervention (15.80,4.40,26) Control (24.00,7.20,8) |  | Stress  Intervention (14.40,5.10,20) Control (22.50,7.20,7) |
|  |  |  |  | same as above two, plus weeks 3, 6, and 8 were facilitated by a licensed clinical counsellor or licensed social worker (n=33) |  |  |  |  | Intervention:  Stress (24.50,5.10,33)  Control: Stress (25.40,5.70,12) |  | Stress  Intervention (15.80,7.00,21) Control (24.00,7.20,8) |  | Stress  Intervention (16.30,5.60,14) Control (22.50,7.20,6) |
| Birney (2016) * | CBT | Yes, depression | USA, Employees with mild to moderate depressive symptoms (n=300) | 6 weeks CBT mobile phone app “MoodHacker” brief daily interactions (n=150) | WLC (n=150) | Tertiary | Depression | Depression (PHQ-9) | Intervention:  Depression (13.20,4.30,150)  Control:  Depression (13.60,3.80,150) | 10 weeks | Depression  Intervention (8.80,5.10,130) Control (9.50,5.00,141) | Nil | not measured |
| Bostock (2016) * | CBT | No | USA, general office-based employees who report poor sleep (n=270) | 8-week access to online Sleepio.com. Animated virtual therapist (‘‘The Prof’’) and tailored by the program’s algorithms to everyone’s characteristics, personal goals, sleep diary data, and progress. Support by system-generated email/SMS prompts and access to a moderated online community (n=135) | WLC (n=135) | Universal | Insomnia | Depression (PHQ-2), Anxiety (GAD-2) | Intervention:  Depression (1.57,0.11,135) Anxiety (2.32,0.13,135)  Control: Depression (1.44,0.12,135) Anxiety (2.16,0.13,135) | 2 months | Depression  Intervention (1.38,0.13,98) Control (1.30,0.12,116)  Anxiety Intervention (1.59,0.15,98) Control (1.80,0.14,116) | nil | not measured |
| Dyrbye (2016) * | Positive Psychology | No | USA, Practicing Physicians (n=290) | 6-week online intervention one domain per week each took less than 5 mins to complete, based well-being, positive psychology, and mindfulness (n=145) | WLC (n=145) | Universal | Well-being | Depression (PRIME MD) | Intervention:  Depression (145)  Control: Depression (145) | 3 months | Depression Intervention (131) Control (134) | nil | not measured |
| Ebert (2016) *^ (a) | Stress Mx | Yes, High stress | Germany, highly stressed employees from health insurance companies (n=264) | 7-week online intervention GET.ON Stress. Weekly modules 45-60 mins each. With daily stress diaries. eCoach (psychologist) sends reminders and feedback and text messages (n=132) | WLC (n=132) | Indicated | Stress | Stress (PSS-10), Depression (CES-D), Anxiety (HADS-A) | Intervention:  Stress (25.70,5.70,132) Depression (25.10,9.31,132) Anxiety (11.40,3.40,132)  Control: Stress (26.10,4.10,132) Depression (23.90,8.30,132) Anxiety (11.30,3.60,132) | 7 weeks | Stress  Intervention (18.10,5.70,119) Control (23.40,5.40,130)  Depression  Intervention (16.10,8.70,119) Control (21.40,9.10,130)  Anxiety  Intervention (8.00,3.70,119) Control (9.90,3.80,130) | 6 months | Stress  Intervention (17.50,6.70,109) Control (21.80,6.70,129)  Depression  Intervention (15.20,9.00,109) Control (20.20,10.00,129)  Anxiety  Intervention (7.20,3.70,109) Control (9.30,4.20,129) |
| Ebert (2016) *^ (b) | Stress Mx | Yes, High stress | Germany, highly stressed employees from health insurance companies (n=264) | 7-week online intervention GET.ON Stress. Eight modules 45-60 mins each. With daily stress diaries. eCoach (psychologist) sends reminders and feedback and text messages (n=132) | WLC (n=132) | Indicated | Stress | Stress (PSS-10), Depression (CES-D), Anxiety (HADS-A) | Intervention:  Stress (25.21,4.59,132) Depression (23.17,9.27,132) Anxiety (10.65,3.35,132)  Control: Stress (25.31,4.16,132) Depression (24.27,8.39,132) Anxiety (11.12,3.25,132) | 7 weeks | Stress  Intervention (18.79,5.85,112) Control (23.33,5.66,128)  Depression  Intervention (17.69,8.21,112) Control (22.46,9.17,128)  Anxiety  Intervention (7.67,3.37,112) Control (10.38,3.45,128) | 6 months | Stress  Intervention (17.05,5.81,97) Control (22.24,6.46,122)  Depression  Intervention (15.52,7.05,97) Control (22.75,9.78,122)  Anxiety  Intervention (6.86,3.28,97) Control (10.04,3.92,122) |
| Heber (2016) * ^ | Stress Mx | Yes, High stress | Germany, mainly recruited by a large health insurance company of highly stressed employees (n=264) | 7 sessions. 1-2 sessions per week. web-based Internet stress management interventions (iSMI) problem solving, emotion regulation strategies + booster session (n=132) | WLC (n=132) | Indicated | Stress | Stress (PSS10), Depression (CES-D), Anxiety (HADS) | Intervention:  Stress (25.90,3.90,132) Depression (23.34,8.50,132) Anxiety (11.20,3.30,132)  Control: Stress (25.15,3.96,132) Depression (23.77,7.60,132) Anxiety (10.70,3.40,132) | 7 weeks | Stress  Intervention (17.90,6.17,132) Control (22.90,6.10,132)  Depression  Intervention (15.60,9.10,132) Control (21.40,8.80,132)  Anxiety  Intervention (7.80,3.90,132) Control (10.30,3.50,132) | 6 months | Stress  Intervention (16.08,6.00,132) Control (22.10,5.80,132)  Depression  Intervention (13.80,7.70,132) Control (31.50,8.50,132)  Anxiety  Intervention (6.73,3.40,132) Control (9.65,3.60,132) |
| Hersch (2016) * | Stress Mx | No | US, general nurses, and nurse managers employed within five public hospitals (n=104) | online intervention BREATHE with open access for 3 months. 7 modules including, identifying, and managing stress, coping skills and mental health information (n=52) | WLC (n=52) | Universal | Nurses Stress | Stress (NSS) | Intervention:  Stress (2.24,0.46,52)  Control: Stress (2.27,0.43,52) | 3 months | Stress  Intervention (2.07,0.38,52) Control (2.35,0.49,52) | nil | not measured |
| Imamura (2016) * | CBT | Both, no moderate and high depression | Japan, general managers, and employees (n=1,236) | 4-week internet access to UTSMed composed of text and illustrations; no video or audio narration was used. The website consisted of about 90 pages, with around 800 Japanese characters per page no depression (n=276) | WLC no depression (n=285) | Tailored | Depression | Depression (BDI-II), Distress (K6) | Intervention:  Depression (7.10,6.00,276) Stress (1.70,1.50,276)  Control:  Depression (7.00,5.00,285) Stress (1.70,1.50,285) | 1 month | Depression  Intervention (7.10,6.80,236) Control (6.10,5.50,254)  Stress Intervention (3.30,3.00,236) Control (2.80,2.80,254) | 4 months | Depression  Intervention (5.80,6.20,211) Control (5.90,5.90,248)  Stress Intervention (2.90,3.00,211) Control (3.20,3.80,248) |
|  |  |  |  | as above, moderate depression (n=291) | as above, moderate depression (n=290) |  |  |  | Intervention:  Depression (17.80,9.30,291) Stress (8.90,3.70,291)  Control:  Depression (18.90,9.60,290) Stress (9.50,3.70,290) |  | Depression  Intervention (16.20,10.20,254) Control (17.20,10.20,263)  Stress Intervention (8.30,4.60,254) Control (8.60,4.40,263) |  | Depression  Intervention (14.90,10.60,231) Control (16.80,10.40,251)  Stress Intervention (8.00,4.70,231) Control (8.70,4.60,251) |
|  |  |  |  | as above, high depression (n=51) | as above, high depression (n=43) |  |  |  | Intervention:  Depression (24.80,14.80,51) Stress (12.40,5.70,51)  Control:  Depression (25.40,12.70,43) Stress (11.90,5.70,43) |  | Depression  Intervention (19.20,13.10,41) Control (24.30,12.70,42)  Stress Intervention (9.90,5.60,41) Control (11.80,5.60,42) |  | Depression  Intervention (21.20,14.20,39) Control (20.80,13.70,40)  Stress Intervention (10.30,6.40,39) Control (10.40,5.90,40) |
| Jonas (2016) * ^ | CBT | Yes, Burnout | Germany, employees with burnout (n=59) | a 4 week online-intervention for burnout and work-related stress “Beratung Hilft” (“counselling helps”). Structured and therapist guided. Daily stress diary. Contact with trained counsellor and guided by a trained psychotherapist (n=18) | WLC (n=21) | Indicated | Burnout | Depression, Anxiety & Stress (DASS-21) | Intervention:  Stress (18.90,10.10,18) Depression (16.70,9.20,18) Anxiety (7.80,7.50,18)  Control: Stress (19.60,10.90,21) Depression (17.40,12.60,21) Anxiety (9.00,10.20,21) | 3 months | Stress  Intervention (13.00,13.10,15) Control (18.60,10.70,21)  Depression  Intervention (7.80,12.30,15) Control (17.00,12.80,21)  Anxiety  Intervention (5.20,7.30,15) Control (9.30,8.60,21) | 6 months | Stress  Intervention (12.50,14.30,13) Control not measured  Depression  Intervention (9.10,11.10,13) Control not measured  Anxiety  Intervention (3.20,9.90,13) Control not measured |
|  |  |  |  |  |  |  |  |  |  |  |  | 12 months | Stress  Intervention (10.60,37.10,9) Control not measured  Depression  Intervention (5.60,9.80,9) Control not measured  Anxiety  Intervention (4.00,12.10,9) Control not measured |
| Beiwinkel (2017) *^ | CBT | Yes, depression | Germany, depressed health insurance employees who are at risk of sick leave with depression (n=180) | “HelpID” is a 12-week, Web-based program based on CBT, awareness training, and systemic counselling. 12 weekly sessions. Each lasted 30 to 45 mins. Weekly reminder emails. The program had a guided format with clinical psychologist contact upon request (n=100) | WLC + Info (n=80) | Tertiary | Depression | Depression (PHQ-9 & BDI) | Intervention:  Depression (11.54.4.35,100)  Control: Depression (10.56,4.53,80) | 3 months | Depression Intervention (6.51,2.87,44) Control (7.76,3.63,44) | Nil | not measured |
| Boß (a) (2017) *, (b) (2017) *^ | CBT | No | Germany, general employees from health insurance companies (n=434) | 5-week web-based intervention GET.ON with one module per week. Each module includes tools to control drinking behavior, and an emotion regulation training Received an unguided self‐help version (n=146) | WLC (144) | Universal | Alcohol consumption | Depression, Anxiety & Stress (DASS-21) | Intervention:  Stress (7.33,4.67,146) Depression (5.17,4.71,146) Anxiety (2.42,2.97,146)  Control: Stress (6.72,4.81,72) Depression (4.60,4.50,72) Anxiety (2.31,2.71,72) | 6 weeks | Stress  Intervention (5.10,3.73,110) Control (6.46,4.50,62)  Depression  Intervention (3.77,4.12,110) Control (4.69,4.43,62)  Anxiety  Intervention (1.63,2.28,110) Control (2.22,2.64,62) | 6 months | Stress  Intervention (5.00,4.00,84) Control (6.10,4.43,50)  Depression  Intervention (4.04,3.76,84) Control (4.60,4.27,50)  Anxiety  Intervention (2.04,3.76,84) Control (2.51,2.87,50) |
|  |  |  |  | Identical to the GET.ON intervention above plus additional adherence‐focused guidance by eCoaches (trained psychologist) (n=144) |  |  |  |  | Intervention:  Stress (6.64,4.80,144) Depression (4.96,4.73,144) Anxiety (1.90,2.42,144)  Control: Stress (6.72,4.81,72) Depression (4.60,4.50,72) Anxiety (2.31,2.71,72) | 6 weeks | Stress  Intervention (5.13,3.40,106) Control (6.46,4.50,61)  Depression  Intervention (3.30,3.10,106) Control (4.69,4.43,61)  Anxiety  Intervention (1.20,1.73,106) Control (2.22,2.64,61) | 6 months | Stress  Intervention (4.39,2.92,87) Control (6.10,4.43,50)  Depression  Intervention (3.43,3.30,87) Control (4.60,4.27,50)  Anxiety  Intervention (1.51,1.60,87) Control (2.51,2.87,50) |
| Carolan (2017) *^ | CBT | Yes, High stress | UK, employees from 20 different organisations with elevated stress (n=84) | WorkGuru is an online CBT based, 8 weeks delivered with minimal guidance from a coach. 7 weekly modules (n=28) | WLC (n=28) | Indicated | Engagement | Depression, Anxiety & Stress (DASS-21) | Intervention:  Stress (24.00,9.40,28) Depression (20.20,9.60,28) Anxiety (12.40,8.60,28)  Control: Stress (24.10,8.00,14) Depression (20.50,9.40,14) Anxiety (13.60,8.40,14) | 2 months | Stress  Intervention (19.30,6.60,20) Control (22.40,7.60,12)  Depression  Intervention (15.10,9.90,20) Control (18.00,11.00,12)  Anxiety  Intervention (9.30,6.30,20) Control (12.70,9.60,12) | 4 months | Stress  Intervention (15.90,6.60,23) Control (20.60,8.70,13)  Depression  Intervention (13.80,9.50,23) Control (16.00,9.90,13)  Anxiety  Intervention (7.90,6.90,23) Control (11.00,9.60,13) |
|  |  |  |  | Participants had access to the intervention as described above; plus, access to an 8 week online guided discussion group that was delivered via a bulletin board (n=28) |  |  |  |  | Intervention:  Stress (23.30,7.70, 28) Depression (19.90,10.20,28) Anxiety (10.80,7.40,28)  Control: Stress (24.10,8.00,14) Depression (20.50,9.40,14) Anxiety (13.60,8.40,14) | 2 months | Stress  Intervention (19.80,9.20,17) Control (22.40,7.60,13)  Depression  Intervention (16.00,10.10,17) Control (18.00,11.00,13)  Anxiety  Intervention (10.20,7.70,17) Control (12.70,9.60.13) | 4 months | Stress  Intervention (18.10,7.70,21) Control (20.60,8.70,13)  Depression  Intervention (15.50,8.50,21) Control (16.00,9.90,13)  Anxiety  Intervention (8.80,6.40,21) Control (11.00,9.60,13) |
| Shirotsuki (2017) * | CBT | No | Japan, general office workers in food and beverage manufacturing/sales company (n=87) | 6 weekly online CBT. Participants watched weekly e-learning movie segments (5–10 min long) and read the corresponding sections of the guidebook. Recorded daily mood and weekly homework (n=29) | The control group recorded their mood state every day on a weekly monitoring sheet (homework) (n=29) | Universal | Anxiety & Fatigue | Anxiety (POMS-A), Depression (POMS-D) | Intervention:  Depression (11.96,12.32,29) Anxiety (14.84,8.76,29)  Control: Depression (7.09,8.49,14) Anxiety (10.09,5.23,14) | 6 weeks | Depression  Intervention (11.52,9.65,25) Control (7.83,9.12,12)  Anxiety  Intervention (12.36,6.13,25) Control (11.83,6.55,12) | nil | not measured |
|  |  |  |  | The CBT intervention was the same as above plus participants consumed one bottle of the supplement soft drink (100 ml) every morning throughout the 6 weeks. The drink contained 200 mg of L-carnosine (n=29) |  |  |  |  | Intervention:  Depression (12.04,11.55,29) Anxiety (13.83,7.23,29)  Control: Depression (7.09,8.49,15) Anxiety (10.09,5.23,15) | 6 weeks | Depression  Intervention (9.83,10.64,24) Control (7.83,9.12,11)  Anxiety  Intervention (12.58,5.52,24) Control (11.83,6.55,11) | nil | not measured |
| Zhang (2017) * | Awareness Training | No | Singapore, health-care workers with heart disease (n=80) | The 4-week smartphone-based coronary heart disease prevention programme. Comprised a newly developed mobile app named Care4Heart, a 20-min briefing session, and a daily short message service (SMS) (n=40) | information from the Singapore Heart Foundation (SHF) and the Health Promotion Board (HPB) (n=40) | Universal | Awareness and knowledge of coronary heart disease | Stress (PSS-10) | Intervention:  Stress (17.00,5.30,40)  Control: Stress (15.70,5.20,40) | 1 month | Stress  Intervention (16.10,4.80,40) Control (16.20,5.10,39) | nil | not measured |
| Zwerenz (2017) *^ | Psychoeducation | Yes, depression | Germany, sick-listed employees in inpatient rehabilitation (n=652) | 12-week online program, weekly reflective 45min blogs instructed by therapist. Reminder emails, individualized feedback, audio guided stress management, homework, and forum (n=303) | Active control, regular e-mail reminders to use selected information posted online about stress management and coping (n=329) | Tertiary | Return to Work | Depression (PHQ-9), Anxiety (GAD-7), Distress (PHQ-D) | Intervention:  Depression (8.56,5.94,303) Anxiety (6.75,5.12,303) Stress (7.80,4.41,303)  Control: Depression (8.40,5.52,329) Anxiety (6.29,4.97,329) Stress (7.42,4.21,329) | 3 months | Depression  Intervention (7.96,5.34,216) Control (8.93,5.61,252)  Anxiety Intervention (6.69,4.62,216) Control (7.25,4.93,252)  Stress Intervention (7.19,4.17,216) Control (7.55,4.14,252) | 12 months | Depression  Intervention (7.86,5.31,201) Control (8.84,5.73,224)  Anxiety Intervention (6.35,4.46,201) Control (7.51,4.80,224)  Stress Intervention (7.13,3.96,201) Control (7.74,4.35,224) |
| Bostock (2018) * | Mindfulness | No | UK, general pharmaceutical, and high-tech employees (n=238) | 8-week Headspace smartphone app. 45 Days of daily 10-20 min mindfulness meditation (n=128) | National Health Service online advice for work stress (n=110) | Universal | Well-being | Anxiety & Depression (HADS) | Intervention:  Depression (5.05,3.40,128) Anxiety (9.13,3.90,128)  Control: Depression (5.13,3.20,110) Anxiety (9.36,4.00,110) | 2 months | Depression  Intervention (3.60,3.20,123) Control (5.18,3.50,106)  Anxiety  Intervention (7.44,3.60,123) Control (8.86,3.90,106) | nil | not measured |
| Eriksson (2018) * | Mindful Self-Compassion Program | No | Sweden, practicing psychologists (n=101) | 6 weeks internet-delivered program. Initial instruction video and six steps involving different types of exercises with guided instructions (auditory files) (1) Kind attention, (2) Kind awareness, (3) Loving kindness with oneself and others, (4) Self-compassion—part 1, (5) Self-compassion—part 2, (6) Compassion with others and Quiet Practice Mindfulness exercises, breathing anchor and body scans (n=51) | WLC (n=49) | Universal | Self-Compassion | Stress (PSS) | Intervention:  Stress (25.38,6.99,51)  Control:  Stress (25.12,8.02,49) | 2 months | Stress Intervention (19.43,6.42,40) Control (23.49,8.56,41) | nil | not measured |
| Gollwitzer (2018) * | Mental Contrasting | No | Germany, general nurses (n=129) | MCII online 3 weeks daily. Imagination technique with reflection on "what if" and written thoughts daily. Identifying obstacles in the way. (n=41) | WLC (n=47) | Universal | Stress | Stress (PSQ) | Intervention:  Stress (2.33,0.57,41)  Control:  Stress (2.58,0.58,24) | 1 month | Stress Intervention (2.13,0.60,33) Control (2.55,0.68,19) | nil | not measured |
|  |  |  |  | IIMCII the same as above plus the setting in concrete of times to conduct the daily reflections with follow-up on if completed (n=41) |  |  |  |  | Intervention:  Stress (2.40,0.46,41)  Control:  Stress (2.58,0.58,23) |  | Stress Intervention (2.33,0.45,34) Control (2.55,0.68,19) |  |  |
| Hamamura (2018) # | CBT | Yes, High stress | Japan, stressed marketing company employees (n=557) | 4-week smartphone app called “ jibun kiroku ” [41], focuses on self-monitoring and awareness of negative thoughts, daily activities, and daily mood. Users record their daily activities on an hourly basis, and evaluate the quality of their sleep, mood, and energy level (n=306) | WLC (n=251) | Indicated | Stress | Stress (K6), Depression (CES-D), Anxiety (STAI) | Intervention:  Stress (7.43,4.80,306) Depression (19.62,8.81,306) Anxiety (49.91,8.29.306)  Control: Stress (7.40,4.27,251) Depression (19.44,7.75,251) Anxiety (49.83,7.30,251) | 1 month | Stress  Intervention (8.08,4.75,248) Control (7.98,4.76,224)  Depression  Intervention (21.59,9.32,248) Control (20.21,8.24,224)  Anxiety  Intervention (50.53,8.64,248) Control (49.57,6.80,224) | nil | not measured |
| Imamura (2018) *^ | CBT | No | Japan, general telecommunication employees (n=706) | 6 week, 6-lesson, Web-based CBT training course 30-minute lesson per week. Voluntary homework. Reminder emails. Participants who submitted their homework received feedback from trained clinical psychologists (n=353) | WLC (n=353) | Universal | Depression | Depression (BDI), Psychological Distress (K6) | Intervention:  Stress (6.12,4.70,353) Depression (12.46,8.44,353)  Control: Stress (6.22,4.51,353) Depression (12.71,9.14,353) | 3 months | Stress  Intervention (5.59,4.83,237) Control (6.22,4.50,304)  Depression  Intervention (10.28,8.40,237) Control (12.39,9.03,304) | 6 months | Stress  Intervention (5.46,4.51,246) Control (6.55,5.18,312)  Depression  Intervention (9.87,7.97,246) Control (12.48,9.77,312) |
|  |  |  |  |  |  |  |  |  |  |  |  | 12 months | Stress  Intervention (5.67,4.98,224) Control (6.57.4.71,289)  Depression  Intervention (10.56,9.48,224) Control (12.71,9.95,289) |
| Lilly (2018) * | MBSR | No | USA and Canada, general emergency telecommunication employees (n=323) | 7-week online intervention (Destress 9-1-1), 1 module per week each took about 30 min including an introduction video and texts and a moderated discussion board. Expectations for outside practice up to 45 mins daily mindfulness homework.  Guided audio, the online training system tracked number of sessions completed (n=163) | WLC (n=160) | Universal | Stress | Stress (C-SOSI) | Intervention:  Stress (57.70,30.40,160)  Control:  Stress (52.10,27.60,159) | 2 months | Stress Intervention (49.70,27.20,110) Control (54.30,30.50,130) | 3 months | Stress Intervention (50.70,28.00,86) Control (52.30,30.50,100) |
| Mistretta (2018) * | MBRT | Yes, High stress | USA, general Hospital employees with at least mild stress levels (n=38) | 6-week smartphone resilience app. Modules included sleep, happiness, positivity, energy, focus and productivity (n = 23) | WLC (n=15) | Indicated | Depression, Anxiety, Stress & Well-being | Depression, Anxiety & Stress (DASS-21) | Intervention:  Stress (7.96,3.36,23) Depression (4.43,3.22,23) Anxiety (4.43,3.22,23)  Control: Stress (7.53,3.38,15) Depression (5.20,4.31,15) Anxiety (4.33,3.42,15) | 6 weeks | Stress  Intervention (6.43,2.83,23) Control (7.13,3.29,15)  Depression  Intervention (3.91,2.66,23) Control (4.53,3.72,15)  Anxiety  Intervention (3.00,3.18,23) Control (2.73,2.79,15) | 3 months | Stress  Intervention (6.17,3.02,23) Control (6.60,2.85,15)  Depression  Intervention (3.52,2.79,23) Control (4.53,3.62,15)  Anxiety  Intervention (2.78,2.73,23) Control (3.67,3.35,15) |
| Oishi (2018) * | CBT | No | Japan, general schoolteachers (n=240) | 12-week online CBT program “Mind Skill Up Training" 7 modules plus 1 group session, includes homework, mood tracking, audio, and visual narrator, 6 reminder emails (n=120) | WLC (n=120) | Universal | Cognitive flexibility | Distress (study specific questions) | Intervention:  Stress (6.03,1.88,120)  Control: Stress (6.28,2.14,120) | 3 months | Stress Intervention (5.24,1.64,117) Control (6.15,1.93,99) | nil | not measured |
| Persson Asplund (2018) *^ | Stress Mx | Yes, High stress | Sweden, distressed middle managers employed in healthcare, education sector, IT, or communications (n=117) | The iSMI consisted of 8 weekly modules. 2–3 hours per week to complete. Participants in the iSMI received weekly personalized written feedback via email from a coach (psychologist) (n=59) | Active control group provided a similar attentional focus (e.g., weekly mail contact, homework assignment) as to the iSMI group. Had access to a moderated discussion forum with other users (n=58) | Indicated | Stress | Stress (PSS-14) Depression (MADRS-S) | Intervention:  Depression (15.71,6.35,59) Stress (33.22,5.27,59)  Control: Depression (15.76,4.99,58) Stress (33.09,5.34,58) | 2 months | Depression  Intervention (9.49,5.11,42) Control (14.64,6.78,51)  Stress Intervention (24.39,5.95,42) Control (29.11,6.70,51) | 6 months | Depression  Intervention (10.64,7.17,38) Control (12.59,6.57,44)  Stress Intervention (22.52,7.22,38) Control (26.61,6.80,44) |
| Querstret (2018) * | MBCT | No | UK, general employees (n=118) | 4-week online intervention, digital Mindfulness-Based Cognitive Therapy (MBCT) programme (n=60) | WLC (n=58) | Universal | Stress | Stress (PSS-10), Depression (PHQ-9), Anxiety (GAD-7) | Intervention:  Stress (24.55,5.53,60) Depression (11.10,6.24,60) Anxiety (10.43,4.96,60)  Control: Stress (24.22,5.79,58) Depression (9.91,5.93,58) Anxiety (8.98,5.32,58) | 3 months | Stress  Intervention (14.57,5.45,45) Control (22.41,7.00,42)  Depression  Intervention (4.10,4.10,45) Control (9.28,5.50,42)  Anxiety  Intervention (4.34,3.94,45) Control (9.19,4.93,42) | nil | not measured |
| Song (2018) * | Health Surveillance Model | No | Japan, general employees (n=1,526) | 16-week smartphone application “Karada-no-kimochi”. The user can record their menstrual dates, basal body temperatures, and their mental and physical disorders. It provides information regarding the condition in the stage of menstrual cycle and recommends appropriate changes in lifestyle, including recommended food and exercises to alleviate symptoms.    Users record their mental and physical disorders (headache, stomach-ache, irritation, depressed mood, etc.) using the “Symptom’s stamp”. (n=612) | WLC (n=914) | Universal | Labor productivity, incidence of diseases, and depression | Depression (PHQ-9) | Intervention:  Depression (14.10,4.70,612)  Control:  Depression (14.20,4.84,914) | 1 month | Depression  Intervention (13.9,4.08,462) Control (14.1,4.41,760) | 2 months | Depression  Intervention (13.6,3.95,433) Control (14.3,4.24,703) |
|  |  |  |  |  |  |  |  |  |  |  |  | 3 months | Depression  Intervention (13.8,3.87,415) Control (14.2,4.22,697) |
| Coelhoso (2019) *^ | Mindfulness meditation | No | Brazil, female private hospital employees (n=490) | 8-week well-being mobile app consists of an 8-week program divided into 2 4-week modules. 4 classes per week. Each class contained a brief theoretical portion and a 15-min guided practice. Twice a week, participants were asked to write reflections in a gratitude journal 20min per week. They were told to do the activities whenever and wherever it felt most appropriate, pop ups, notifications, and feedback scores (n=250) | The control app had the same features as the well-being mobile app, including a menu, a tutorial, a profile page, evaluations, pop-up messages, and push notifications. Same period as the intervention, participants had to answer 4 assessments per week 20-min interval (n=240) | Universal | Stress | Stress (PSS-10) | Intervention:  Stress (21.75,13.69,250)  Control: Stress (22.78,12.09,240) | 2 months | Stress Intervention (15.60,8.94,116) Control (20.15,8.71,110) | nil | not measured |
| Stratton (2019) * | Disclosure Decision Aid Tool | Yes, any mental health condition | Australia, employees with a mental health condition, recruited from a range of industries (n=107) | READY online disclosure decision aid tool. 2 weeks access, 7 modules, approximately 60 mins to complete (n=53) | Information provided about disclosure on leading NGO website (n=54) | Tertiary | Decisional Conflict | Depression (PHQ-9), Stress (PSS-10) | Intervention: Depression (16.40,7.30,53) Stress (25.30,6.60,53)  Control: Depression (13.60,5.70,54) Stress (23.90,5.00,54) | 2 weeks | Depression  Intervention (15.90,4.30,41) Control (14.20,2.90,42)  Stress Intervention (24.90,2.80,41) Control (24.40,1.90,42) | 6 weeks | Depression  Intervention (14.50,5,00,25) Control (14.30,3.60,20)  Stress Intervention (24.20,3.90,25) Control (23.80,5.00,20) |
| Weber (2019) * | Behavior change | No | European businesses in Germany, England, and Northern Ireland from the private and public sector (n=532) | 4-week mobile application "Kelaa Mental Resilience App". 28 sessions daily. 2 key modules (1) measuring behavior, cognitions, and emotions (tracking module, Track mood and health, gives feedback) and (2) providing psycho-educational content, six to seven “daily sessions” (each about 2–4 min to read) (n=210) | WLC (n=322) | Universal | Stress | Stress (COPSOQ II) | Intervention:  Stress (3.00,0.76,199)  Control: Stress (3.01,0.73,299) | 1 month | Stress Intervention (2.53,0.82,117) Control (2.79,0.81,241) | 6 weeks | Stress Intervention (2.46,0.80,111) Control (2.57,0.81,225) |
| Deady et al (2020) * | Behavior Activation and Mindfulness | No | Australia, employees working in male-dominated industries (n=2257) | HeadGear is a smartphone application - 30-day intervention in which users complete one ‘challenge’ daily (5–10 minutes/day). Features include risk calculator with personalized feedback. Other components include a mood tracker, a toolbox of skills, and support service helplines (n=1131) | The active control - a smartphone application with an identical design as HeadGear with restricted access to the risk calculator and mood tracker daily over a 30-day period (n=1144) | Universal | Depression | Depression (PHQ-9) | Intervention:  Depression (6.90,3.60,1128)  Control: Depression (6.90,3.50,1143) | 5 weeks | Depression Intervention (5.27,3.70,470) Control (5.87,4.04,618) | 3 months | Depression Intervention (4.95,4.26,465) Control (5.53,4.37,580) |
| Key: * Randomized controlled trial # Controlled Trial $ Pre-intervention post-intervention ^ Guided Stress Mx = stress management, ACT = acceptance and commitment therapy, CBT = Cognitive behavioural therapy, MBSR = Mindfulness based stress reduction, MBRT = Mindfulness based resilience training, MBST = Mind-Body Skills Training, MBCT = Mindfulness based cognitive therapy,  Questionnaires used: Hospital Anxiety Depression Scale (HADS), Brief Job Stress Questionnaire (BJSQ), Centre of Epidemiologic Studies Depression scale (CES-D), Beck Anxiety Inventory (BAI), Visual Analogue Scale (VAS), Beck’s Depression Inventory (BDI-21), Brief Symptom Inventory (BSI), Kessler 6 (K6), Depression Anxiety Stress Scales DASS, 21 & 42), Patient Health Questionnaire-9 (PHQ-9), Work and Social Adjustment Scale (WSAS), Perceived Stress Scale (PSS, 10 &14), Patient Satisfaction Questionnaire (PSQ), General Health Questionnaire (GHQ-12), Job satisfaction Survey (JSS), Job Stress Burnout (JSBQ), State-Trait Personality Inventory (STPI), Measure du Stress Psychologique (MSP), State Trait Anxiety Inventory (STAI), Job Content Questionnaire (JCQ), Brief Coping Orientation to Problems Experienced (COPE), WHO-Five Well-being Index (WHO-5), Who-subjective wellbeing (WHO-SUBI), Stress Warning Signals Scale (SWS), Visual Analog Scale (VAS), Freiburg Mindfulness Inventory (FMI), Recovery Experience Questionnaire (RECQ), Flourishing Scale (FS), Nurses Work Functioning Questionnaire (NWFQ), Dutch Questionnaire on the Experience and Evaluation of Work (QEEW), Four-Dimensional Symptoms Questionnaire (4DSQ), Work Ability Index (WAI), Attributional Style Questionnaire (ASQ),Bergen Burnout Indicator (BBI-15), General Symptom Index (GSI), Acceptance and Action Questionnaire-2 (AAQ-2), Effort-Reward Imbalance (ERI), Health and Work Performance Questionnaire (HPQ), Health-related quality of life (SF-12), Maslach Burnout Inventory (MBI) & emotional exhaustion (MBI-EE) & exhaustion (MBI-EX), and professional efficacy (MBI-PE), Penn State Worry Questionnaire (PSWQ), General Self-Efficacy Scale (GSE), Behavioral Activation for Depression Scale (BADS), Automatic Thoughts Questionnaire-Revised (ATQ-R), Work Limitations Questionnaire (WLQ), Workplace Outcome Suite (WOS), Clinical Outcomes in Routine Evaluation (CORE10), EuroQol (EQ-5D), Service Receipt Inventory (CSRI), Utrecht Work Engagement Scale (UWES), Emotion Regulation Skills Questionnaire (ERSQ), Client Satisfaction Questionnaire (CSQ-8), Medical Technology Assessment Cost Questionnaire for Psychiatry (TiC-P-G), Insomnia Severity Index (ISI), Cognitive and Affective Mindfulness Scale–Revised (CAMS-R), The Pittsburgh Sleep Quality Index (PSQI), Detecting Alcoholism Cutting down, Annoyance by criticism, Guilty feeling, and Eye-openers (CAGE), Wellbeing at Work (IWP), Self-efficacy Scale (SES), Social Problem-Solving Inventory (SPSI), Attitudes Towards Help Seeking (ATHS), Symptoms of Distress Scale (SDS), Stress Relief Strategies Questionnaire (SRSQ), Beck Anxiety Inventory (BAI), Positive and Negative Affect Schedule (PNAS), International Physical Activity Questions (IPAQ), Subjective Units of Distress (SUDS), Nutritional Assessment and Self-efficacy Evaluation (NASE), Weight Control Assessment scale (WQAS), Eating Self-Efficacy Scale (ESES), Godin Leisure-Time Exercise Questionnaire (GLTEQ), Brief Scales for Coping Pro le (BSCP), Positive Affect Schedule Negative Affect Schedule (PANAS), Selbsteinschätzung Emotionaler Kompetenzen (EMO-CHECK/SEK-27), Shirom Vigor Scale (SVS), Connor-Davidson Resilience Scale (CD-RISC), Five Facets of Mindfulness Questionnaire (FFMQ), Multifactor Leadership Questionnaire (MLQ), Mindfulness Attention Awareness Scale (MAAS), RAND Corporation's Medical Outcomes Study Short Form-36 (SF-36), The World Health Organization Quality of Life – BREF (WHOQOL-BREF), Authentic Happiness Inventory (AHI), Wechsler Adult Intelligence Scale - Fourth Edition (WAIS-IV), Workplace Productivity (LOC), Job Satisfaction (JSS), Intention to Quit (ITQ), Professional Self-Esteem (PSES), Quality of life scale (QOLS), The Structure of Psychological Well-being (TSPW), Assertive Mind Scale (AMS), Assertion Check List (ACL), Career Identity Scale (CIS-21), Profile Mood Scale (POMS), Somatosensory Amplification Scale (SSAS), Medical Symptom Checklist (MSCL), General Self Efficacy Scale (GSES), Standard Units of Alcohol (SUA), Irritation Scale (IS), Effort Reward Imbalance Questionnaire – Short Form (ERI‐SF), Nursing Stress Scale (NSS), Nurses Job Satisfaction Scale (NJSS), The brief encounter psychosocial instrument (BEPSI), Brief Encounter Psychosocial Instrument (BEPSI-K), Social Readjustment Rating Scale (SRRS), Korean Resilience Quotient-53 (KRQ-53), International Physical Activity Questionnaire (IPAQ), Mini Dietary Assessment (MDA), Fagerström Test for Nicotine Dependence (FTND), Alcohol Use Disorders Identification Test (AUDIT), The Warwick-Edinburgh Mental Wellbeing Scale (WEMWBS), Whitehall II Study Questionnaire (WII), Social Support At Work (SSAW), Self-Compassion Scale (SCS), MBI-Human Services Survey (MBI-HSS), Patient Reported Outcomes Measurement Information System (PROMIS), Brief Serenity Scale (BSS), System Usability Scale (SUS), Professional Quality of Life-Revision IV (ProQOL), Outcome Questionnaire 45 (OQ-45), Connor-Davidson Resilience Scale (CD-RISC), Provider Resilience Questionnaire (PRQ), World Health Organization Health and Work Performance (WHO-HPQ), Work-Related Acceptance and Action Questionnaire (WAAQ), personal and work community well-being (P-TyHy), Self-Regulation Questionnaire (SRQ), Karasek's Job Content Questionnaire (KJCQ), The Shirom-Melamed Burnout Questionnaire (SMBQ), The Work Experience Measurement Scale (WEMS), Supervisor Scale (SS), Health and Safety Executive (HSE), Coping With Stress (CWS), Tobacco Abstinence Self-efficacy Scale (TASS), Godin Leisure-Time Exercise Questionnaire (GLTEQ), Physician Job Satisfaction Scale (PJSS), Primary Care Evaluation of Mental Disorders (PRIME MD), NEO Personality Inventory (NEO PI), Daily Drinking Questionnaire (DDQ), Japanese Negative Mood Regulation Scale (JNMRS), Calgary Symptoms of Stress Inventory (C-SOSI), Effort-Reward Imbalance (ERI), Mental Demands subscale (MDS), Technostress creators’ Scale (TCS), Health Behavior Questionnaire (HBQ), Coping Self-Efficacy Scale (CSES), Interpersonal Reactivity Index (IRI), Heart Disease Fact Questionnaire-2 (HDFQ-2), Behavioural Risk Factor Surveillance System (BRFSS), Sleep Condition Indicator (SCI), The Work Productivity and Impairment questionnaire (WPAI), Perseverative Thinking Questionnaire (PTQ), Dietary Behavioral Intentions (DBI), Dietary Self-Efficacy (DSE), Dietary Stage of Change (DSC), Weight Stage of Change (WSC), Coping (Brief COPE), Godin Leisure-Time Exercise Questionnaire (GLTEQ), Burnout Screening Scales II inventory (BOSS II), Short Screening Instrument for the Assessment of Need for Occupation Related Treatment in Medical Rehabilitation (SIBAR), Subjective Prognosis of Gainful Employment Scale (SPE), Copenhagen Psychosocial Questionnaire – Revised Version (COPSOQ II), Warwick-Edinburgh Mental Wellbeing Scale (WEMWS), Resilience Scale (RS-13), Work Productivity and Activity Impairment Questionnaire: General Health V2.0 (WPAI:GH), Psychological capital (PsyCap). | | | | | | | | | | | | | |
